# The acoustics of bulge rise and rupture at Strokkur geyser

**DOI:** 10.1007/s00445-025-01876-3

**Published:** 2025-09-13

**Authors:** Julia E. Gestrich, Corrado Cimarelli, David Fee, Antonio Capponi, Caron E. J. Vossen, Markus Schmid

**Affiliations:** 1 Department of Earth and Environmental Sciences, Ludwig-Maximilians-Universität München, Theresienstraße 41, München, 80333 Germany; 2 Alaska Volcano Observatory, Geophysical Institute, University of Alaska Fairbanks, 2156 Koyukuk Dr, Fairbanks, AK 99775 USA

**Keywords:** Geyser, Volcano, Monopole, Acoustic, Infrasound, Fountain

## Abstract

Geysers are natural geothermal features that episodically erupt hot water and steam due to the buildup and release of subsurface vapor bubbles. At Strokkur geyser, Iceland, eruptions begin with the growth of a surface bulge caused by rising bubble clusters, followed by gradual rupture and disintegration into a water fountain. In this study, we investigate the high-resolution acoustic and visual signatures of this initial phase of the eruption, providing detailed insights into the fluid dynamics that govern bulge formation and rupture. While similar eruptive behavior is sometimes observed in low-viscosity volcanic systems, Strokkur offers a uniquely transparent medium in which processes like bubble rise and clustering can be directly observed, providing analogies to otherwise obscured dynamics in lava or mud-dominated settings. We combine low-frequency infrasound and high-frequency audio recordings with high-speed video, using synchronized data to track the evolution of the bulge. The results demonstrate that infrasound effectively detects bulge growth, while the onset of rupture is marked by a rise in audio-frequency amplitude. A monopole model is used to simulate pressure variations during bulge growth. The observed decompression signal is associated with the downward water motion during bulge disintegration. These findings improve our understanding of geyser eruption dynamics and suggest how acoustic monitoring can provide valuable information about subsurface processes in both geysers and volcanoes, such as dome inflation or gas bubble accumulation beneath magma surfaces.

**Supplementary Information:**

The online version contains supplementary material available at 10.1007/s00445-025-01876-3.

## Introduction

Geysers are dynamic geothermal features that have played significant spiritual, economic, and touristic roles throughout history (e.g., Barrick 2010; Byrand 2007; Arriaga 2005). Scientific inquiry into their eruptive mechanisms spans more than two centuries, beginning with the first documented account by Mackenzie (1842). These mechanisms generally involve the geothermal heating of water or pressure changes leading to exsolution and the accumulation of subsurface gas bubbles (Hurwitz and Manga 2017). The subaerial structure around geysers leads to a classification of two types: pool geysers, which erupt from broad, water-filled basins, and cone geysers, which erupt from elevated, cone-shaped formations surrounding their conduits.

Eruption styles vary widely, from brief bursts sometimes preceded by surface water bulging, such as at Strokkur geyser in Iceland (Eibl et al. 2024), to prolonged fountaining that can last for hours, as seen at Pohutu geyser in New Zealand (Nishi et al. 2000) or Lone Star geyser in Yellowstone, USA (Karlstrom et al. 2013). This diversity makes geysers excellent natural laboratories for studying fluid and gas dynamics associated with eruptions.

**Fig. 1:**
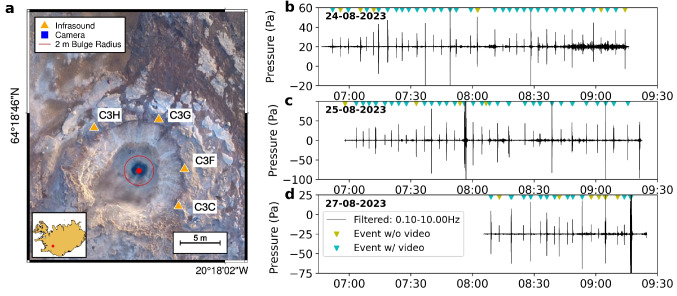
**a** Map of Strokkur geyser pool and surrounding area using the orthomosaic by Walter (2024). The infrasound sensor locations are marked with orange triangles, and the location of the camera used in this study is marked with a blue square. The center of the conduit is marked with a red dot and a circle with an outline of 2 m radius is shown to mark the approximate extent of the bulge. The inset figure is showing a map of Iceland with the location of the geyser marked by a red dot. **b**–**d** Overview of the acoustic waveforms recorded between 24 and 27 August 2023 during which the sensors were located as shown in **a**. The black line shows the waveform filtered in the low infrasound frequency band between 0.1 and 10 Hz. The triangles above the waveform mark the timing of the fountaining events and whether they were captured by video (blue) or not (yellow)

Strokkur geyser, a pool geyser located in Iceland’s Haukadalur Valley, currently erupts every 4 to 10 min (Eibl et al. 2020), sending jets of water and steam up to 20 m high. Its regularity and accessibility make it an ideal site for scientific study. Strokkur’s activity depends on specific hydrothermal conditions: a persistent heat source, ample water supply, and a unique subsurface plumbing system composed of chambers and conduits (e.g., Eibl et al. 2021; Hurwitz and Manga 2017). The geyser’s eruptive behavior has varied historically, ceasing after an earthquake in 1896 and resuming following a reactivation borehole drilled in 1963 (Gudmundsson 2017).

The pool at Strokkur, approximately 7 to 8 m in diameter, is bordered by sinter terraces (Fig. 1a) and fed by a conduit that begins as a roughly 2.2 m in diameter opening and narrows into an elliptical shape with depth (Walter et al. 2020). At depths of 22 to 25 m, deep cavities trap steam bubbles, which expand and trigger eruptions via ascent-driven decompression boiling (Eibl et al. 2021; Walter et al. 2020). Seismic and pressure signals provide insights into these subsurface processes, which are key to understanding Strokkur’s eruptive dynamics (Eibl et al. 2021).

During each eruption cycle at Strokkur, rising gas bubble clusters form a visible water bulge at the surface. As the bulge thins radially, it eventually ruptures, producing a water fountain. These surface changes not only serve as visual eruption precursors but also generate acoustic waves. Similar surface processes have been observed in low-viscosity volcanic settings, including magma surface bulges (Vergniolle and Brandeis 1996; Yokoo et al. 2008), lava fountains (e.g. Gestrich et al. 2022; Lamb et al. 2022), and gas-rich lava lake explosions, such as those at Erebus Volcano (Gerst et al. 2013). Mud volcanoes also exhibit analogous behavior (Rudolph et al. 2024). However, observing such dynamics at volcanoes is significantly more challenging due to the opacity of magma, the rarity of eruptive events, and the danger of approaching active vents. One of the key advantages of studying Strokkur is the transparency of the water. This allows direct visual observation of the bubble cluster dynamics occurring within the bulge similar to laboratory experiments which have provided further insights into the relationship between eruptive fluid dynamics and geophysical signals (e.g., Seyfried and Freundt 2000; James et al. 2008; Lane et al. 2013; Del Bello et al. 2015; Capponi et al. 2017).

Although acoustic sensors have previously been used to detect geyser and hydrothermal activity (Johnson et al. 2013; Cros et al. 2011; Quezada-Reyes 2012; Nishimura et al. 2006), these efforts primarily focused on signal detection and eruption timing. Detailed waveform analysis to characterize acoustic sources has rarely been applied in geyser studies. In contrast, infrasound source characterization is well established in volcanology. The acoustic monopole model developed by Lighthill (1978), for example, has been widely used to describe point-source expansion in transient volcanic explosions (e.g., Woulff 1976; Fee and Matoza 2013; Johnson and Miller 2014; Iezzi et al. 2019), plume dynamics (Yamada et al. 2018), and magma plug (Yokoo et al. 2008) or bubble growth preceding Strombolian-style eruptions (Vergniolle and Brandeis 1996; Gerst et al. 2013; Lyons et al. 2019).

In this study, we combine synchronized high-speed videos and acoustic data to investigate the acoustic signature of water bulge formation and rupture at Strokkur geyser. While high-resolution observations of surface bulges exist, particularly in laboratory settings, this study is the first to directly link visual observations of bubble cluster and bulge dynamics with acoustic signals in geysers. Previous studies in geyser and volcanic contexts have largely relied on inferred dynamics or low-resolution, distant observations. By capturing the eruption’s initiation phase with high spatial and temporal precision, we provide new insights into the coupled visual and acoustic behavior of eruptions. These results help model near-surface dynamics and demonstrate how geysers, due to their accessibility and frequent eruptions, serve as effective analogs for studying eruptive processes relevant to active volcanoes.

## Data

A field campaign was carried out from 24 to 27 August 2023, with the times of recording shown in Fig. 1b–d, totaling 6 h and 14 min. During this time, we deployed four Chaparral M-60 UHP2 infrasound microphones with a flat response from 0.05 to 200 Hz. The microphones were sampled at 400 Hz using DiGOS DATA-CUBE digitizers which had an external GPS antenna for accurate timing. The infrasound microphones were located in a semicircle around the geyser pool (see orange triangles in Fig. 1a) with an approximate distance between the sensors and the center of the pool of 7.5 m. The microphone locations were chosen based on the predominant direction of the falling water and the geyser’s outflow, both of which were toward the southwest, preventing a more circular configuration.

**Fig. 2:**
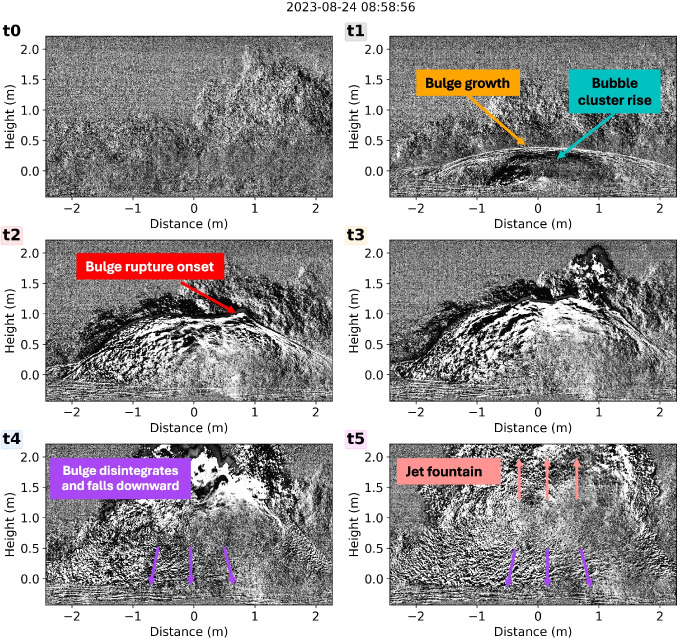
Video frames with applied background subtraction showing different times of the bulge growth (**t0**, **t1**), rupture (**t2**), and disintegration (**t3**–**t5**). The corresponding timing of **t1**–**t5** within the video and acoustic signal is shown in Fig. 8. Time **t0** corresponds to the start of the video before the bulge growth starts. The text in the colored boxes and arrows describe the dynamics during the frame and are also found in Fig. 5

A Chronos 2.1-HD high-speed camera (500 fps; 1920 $\times$ 1080 resolution) with a Nikon ED AF Nikkor 70–300-mm lens at f4–f5.6 was located at a distance of approximately 17.4 m from the center of the pool (see blue square in Fig. 1a). For this camera sensor size, lens focal length, and a distance of 17.4±2 m, the pixel size corresponds approximately to 2.4±0.3  mm. We include this uncertainty to account for the 2D projection of the bulge (approximately 4 m in diameter Eibl et al. 2024) as its outline and center may shift within the camera field of view, as well as slight changes between camera position and lens focal length during the campaign. The total field of view is therefore approximately 4.6 m $\times$ 2.6 m. Video and acoustic data were synchronized using a manual trigger connected to both the high-speed camera and a DiGOS DATA-CUBE digitizer. The videos were recorded on a buffer ring. The pressing and release of the manual trigger changed the voltage on the digitizer, which stopped the recording of the video as a result. The recording of the voltage change allowed each camera trigger event to be simultaneously recorded in the GPS-timed data stream of the DATA-CUBE, ensuring precise synchronization between video and acoustic signals.

In total, 102 events were recorded acoustically during the time of monitoring. On August 24, sensor C3F was moved to a different location after 08:32 a.m. UTC to record a separate geothermal feature. During this time, we will evaluate only the signals from the three remaining sensors. The detection is done by marking the timing of the events during the campaign and cross checking those with the acoustic time series as they are marked by a sharp increase in amplitude across multiple frequency bands (Fig. 1b–d and Supplemental Figs. S1 and S2). Considering all events (including doublets, i.e., two fountains following each other within 30 s), the average inter-event time is 3.52 min (Supplemental Fig. S3), in agreement with the analysis by Eibl et al. (2020). Of the 102 events analyzed, 45 exhibited similar infrasound waveforms, which can be described as an M-shape which will be described and discussed in more detail in “Acoustics.”

Of the 102 monitored events in total, we recorded 73 with the Chronos high-speed video camera to detail the dynamics of the bulge rise and bubble cluster bursting. Of the 45 M-shaped events identified acoustically, 29 of these were used for a detailed video analysis. The other events either lacked video footage or were blurred by the steam between the bulge and the camera.

## Methods

### Video

The high-speed video captures a clear evolution of the bulge growth and disruption which can be generalized into three phases: (1) the water bulge grows smoothly and bubble clusters rise within (Fig. 2t0–t1); (2) the bulge ruptures first at one or multiple small points (Fig. 2t2) which widen over time; (3) the bulge fully disintegrates (Fig. 2t3–t5).

To enhance the visibility of the bulge outline and the bubble cluster within, we remove the static background by subtracting from each frame the third last one. The background subtraction increases the contrast, making the bulge boundaries and rising vapor bubbles more distinct and suitable for tracking (Supplemental Fig. S4 and Fig. 2).

To track the temporal evolution of the bulge, we generate kymograms for each event, a widely used method to visualize the time evolution of height and structures in geysers and volcanic eruptions (e.g. Delle Donne and Ripepe 2012; Lamb et al. 2022; Muñoz et al. 2022; Eibl et al. 2023, 2024). We select a 20-pixels wide vertical window through the center of each frame and average the horizontal pixels to get one grayscale value for each vertical pixel. We do this for each frame and plot them sequentially to generate a kymogram. An example of this is shown for the raw video in Supplemental Fig. S4c and for the video with applied background subtraction in Supplemental Fig. S4d. The time of rupture is picked manually. Due to the rupture being a gradual process, the error is estimated at ± 5 frames which corresponds to ± 0.01 s. The initial rupture point is not always centered in the middle of the bulge and may therefore not be captured by the kymogram, which only represents the vertical centered sector of a video frame.

**Fig. 3:**
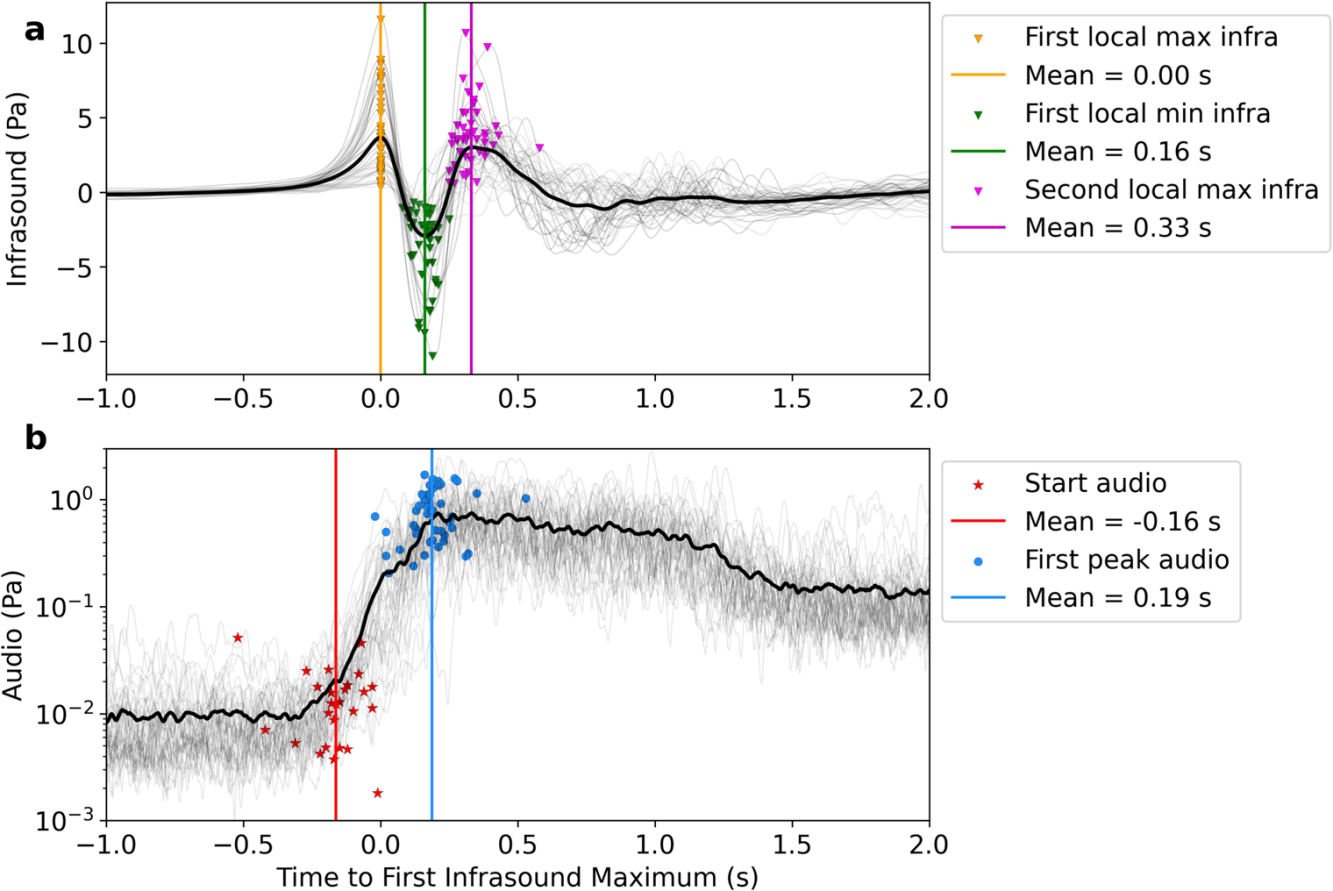
Average infrasound waveform (**a**) and audio envelope (**b**) for the 45 events with an M-shape, aligned by the first infrasound maximum (*t*=0). The thin grey lines each show the individual events and the thick black lines shows the average. The timing and amplitude of the different acoustic parameters are shown in **a**, including the first infrasound local maximum (yellow triangles), following minimum (green triangles), second maximum (purple triangles), and their average timing as vertical lines of the same color. The start of the audio amplitude (red stars) and the first significant increase in audio amplitude (blue circles) with their average timings as vertical lines are shown in **b**

### Acoustics

The acoustic data are filtered in two different frequency bands, 0.1–10 Hz and 10–200 Hz, to capture different scales of dynamics during the eruption. In the subsequent discussion, we refer to these as the infrasound and audio bands, respectively. Cross-correlation analysis reveals zero time lag between station pairs, confirming that the sensors are equidistant from the sound source. Using a moving-window approach, we compute Pearson correlation coefficients between all sensor pairs. The eruptive signal stands out with significantly higher correlation values compared to the surrounding background noise (Supplemental Figs. S5–S8).

To analyze the infrasound signal, we perform waveform stacking by averaging data from all four sensors (Supplemental Fig. S9). The resulting waveforms show very small amplitude variation of only ± 0.16 Pa indicating strong coherence across the array (Supplemental Fig. S10).

For the audio, we calculate the Hilbert transform envelop of the stacked waveform, smoothed by using a 0.02-s rolling mean, in order to evaluate the change in amplitude throughout the event (Supplemental Fig. S9). To quantitatively compare the overall audio amplitude of each event, we calculate the sound pressure level (SPL) using $\textrm{SPL} = 10 \log _{10}\left( \frac{p_{\textrm{rms}}^2}{p_{\textrm{ref}}^2} \right) ,$ where the root mean square pressure is defined as $p_{\textrm{rms}}^2 = \frac{1}{T} \int _0^T p^2(t) \, dt$. Here, $p(t)$ is the stacked and filtered audio pressure, and $p_{\textrm{ref}} = 20\,\mu \textrm{Pa}$ is the reference pressure. We integrate over a time window of $T = 3\,\textrm{s}$, starting 1 s before the first infrasound maximum. This window, shown in Fig. 3, serves as a representative average value for each event.

**Fig. 4:**
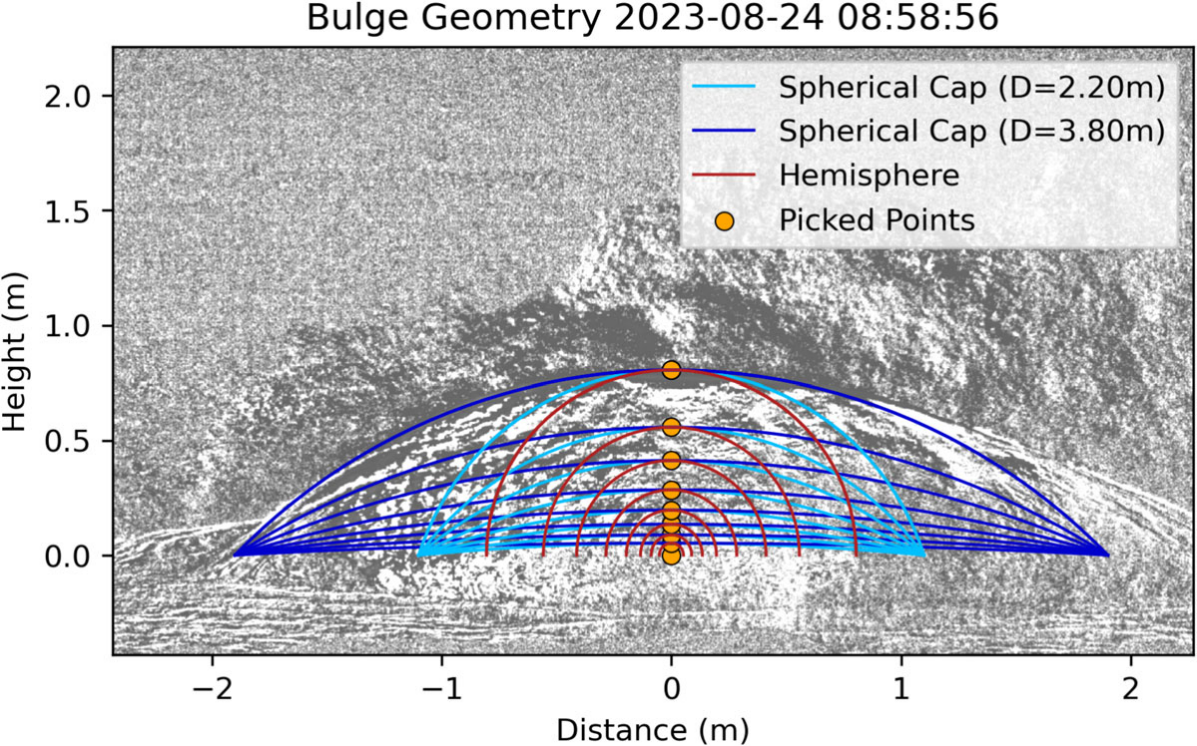
Video frame showing the time of the rupture (background grayscale image) as well as the bulge height points (orange circles). The hemisphere geometry approximation is shown with red lines and the spherical cap approximation is shown for the minimum diameter of 2.2 m in light blue and for the maximum diameter of 3.8 m in dark blue

Several acoustic parameters, including the onset of amplitude rise and key timing features, were identified through manual picking. Although automatic methods, such as STA/LTA and other threshold-based algorithms, were tested, they produced inconsistent results across events due to variability in waveform shape and background noise levels. Manual selection proved to be the most robust and consistent approach. While some uncertainty is inherent in this process—particularly in identifying the precise moment the amplitude exceeds background levels—we estimate a typical timing uncertainty of ± 0.01 s. In most cases, the onset was visually well defined.

The fundamental mechanism that connects a symmetrical volume displacement to the generated pressure perturbations is described by Lighthill (1978) in the form of the monopole acoustic source model. This model has been successfully used to connect subaerial bulge growth dynamics in the volcanic environment (e.g., Vergniolle and Brandeis 1996; Lyons et al. 2019). As the infrasound sensors are in the near-field of the source, this model only serves as a first approximation to explore the potential for estimating the sound from the volume displacement of the bulge. Note that unlike the lava bulges observed by Vergniolle and Brandeis (1996) and Bouche et al. (2010), our high-speed videos show no vibration nor oscillation of the liquid surface before rupturing, which is why we do not use their modification of the model to describe the infrasound observations. Instead, we use the fundamental equation for pressure perturbations from a monopole acoustic source into a half-space by Lighthill (1978). This is purely based on the rate of mass displacement $\dot{q}$ of air with the air density $\rho _{air}$ at the source-receiver distance *r* and an acoustic velocity *c*:

1 $$\begin{aligned} p(t)&= \frac{\dot{q}(t-r/c)}{2\pi r}, \end{aligned}$$

2 $$\begin{aligned}&= \frac{\rho _{air}}{2\pi r} \frac{d^2}{dt^2}V(t-r/c) . \end{aligned}$$

We use two different geometries to approximate the bulge shape based on visual and kymogram observations. The first geometry we assume is a hemisphere. Its volume can be calculated using

3 $$\begin{aligned} V_{hs}&= \frac{2\pi R^3}{3}, \end{aligned}$$

with *R* being the hemisphere radius, or, in our case, the bulge height. The second geometry is a spherical cap which we define by fixed lateral extends of the bulge and changing heights. This means that the actual radius is decreasing with increasing height, but the center of the sphere is moving upwards. The volume of a spherical cap is defined by

4 $$\begin{aligned} V_{sc}&= \frac{\pi }{6} h (3x_w^2+h^2), \end{aligned}$$

where *h* is the height of the bulge and $x_w$ is half of the bulge diameter (or lateral extent). We vary $x_w$ between 1.1 and 1.9 m, corresponding to bulge diameters of 2.2 m and 3.8 m. These values represent the conduit diameter reported by Walter et al. (2020) and Eibl et al. (2024), respectively. The resulting geometry is shown in Fig. 4.

## Results

### Video

**Fig. 5:**
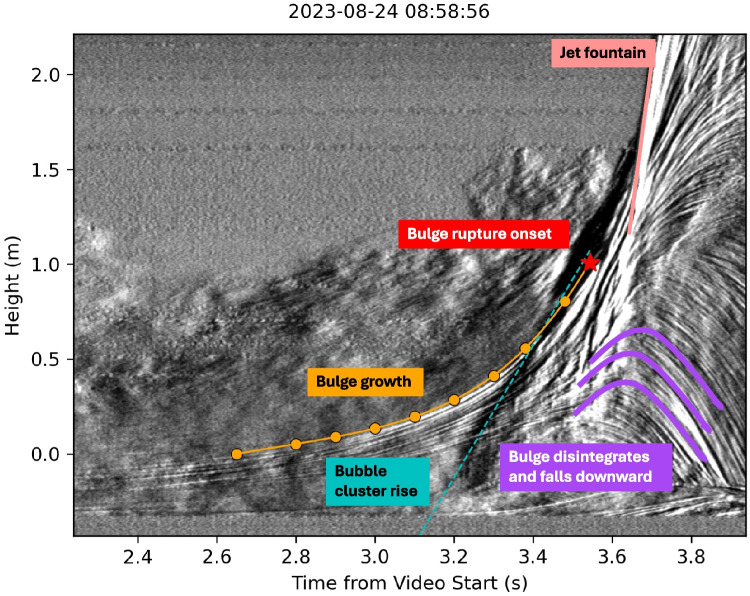
Annotated kymogram of the high-speed video with applied background subtraction. The orange circles indicate the picked points along the line that marks the bulge growth. The orange line is the approximation of the bulge growth function using the picked points and a polynomial of 5th degree. The bubble cluster that rises within the bulge is marked with a dashed blue line. The height and time of the bulge rupture initiation are marked with a red star, and the subsequent jet is marked in light red. The concave purple lines highlight the downward movement and disintegration of the bulge. Note that the three purple lines are meant to describe the general trend of the many concave horizons in their vicinity. The text in the colored boxes and arrows describes the dynamics during the frame and is also found in Fig. 2

Figure 5 shows how different stages, like bulge growth, bubble rise, and disruption, are represented in the kymogram compared to the video frames shown in Fig. 2. In the video (V1_Timings.mp4), the liquid surface rises and forms a bulge shaped as a spherical cap. In the kymogram; this bulge appears as a smoothly emerging line (marked in orange in Fig. 5), which we fit with a polynomial of 5th degree. Hereafter, we will refer to this line as the bulge growth function. This method allows us to resample the bulge growth function for comparison with the acoustic measurements and other quantitative analyses.

In the videos, clusters of rising gas bubbles are visible below the bulge surface (Fig. 2). In the kymogram, the ascent of these bubble clusters is represented by an approximately linearly rising black line (marked in cyan in Fig. 5). The intersection between the bulge growth function and bubble rise line roughly marks the bulge rupture. We also observe that sometimes the bubble cluster reaches the top of the bulge and rises together with it before bursting. We refer to this as bubble lingering.

Generally, the initially small rupture point of the liquid film gradually widens radially (see timing t2 in Video V1_Timings.mp4) producing ripples on the bulge surface (see timing t3 in Video V1_Timings.mp4). After bubble bursting, the mostly upward directed jet is represented by a roughly straight steep interface (light red line in Fig. 5).

During rupturing, the bulge continues to grow (Video V1_Timings.mp4). However, its continuous growth is more difficult to quantify due to the superposition of the burst and the jet dynamics. Following its rupture, the bulge fragments in all directions and “sheds” its water film also downwards. The downward motion of the disintegrating bulge film is represented by overturning horizons (purple lines in Fig. 5).

**Fig. 6:**
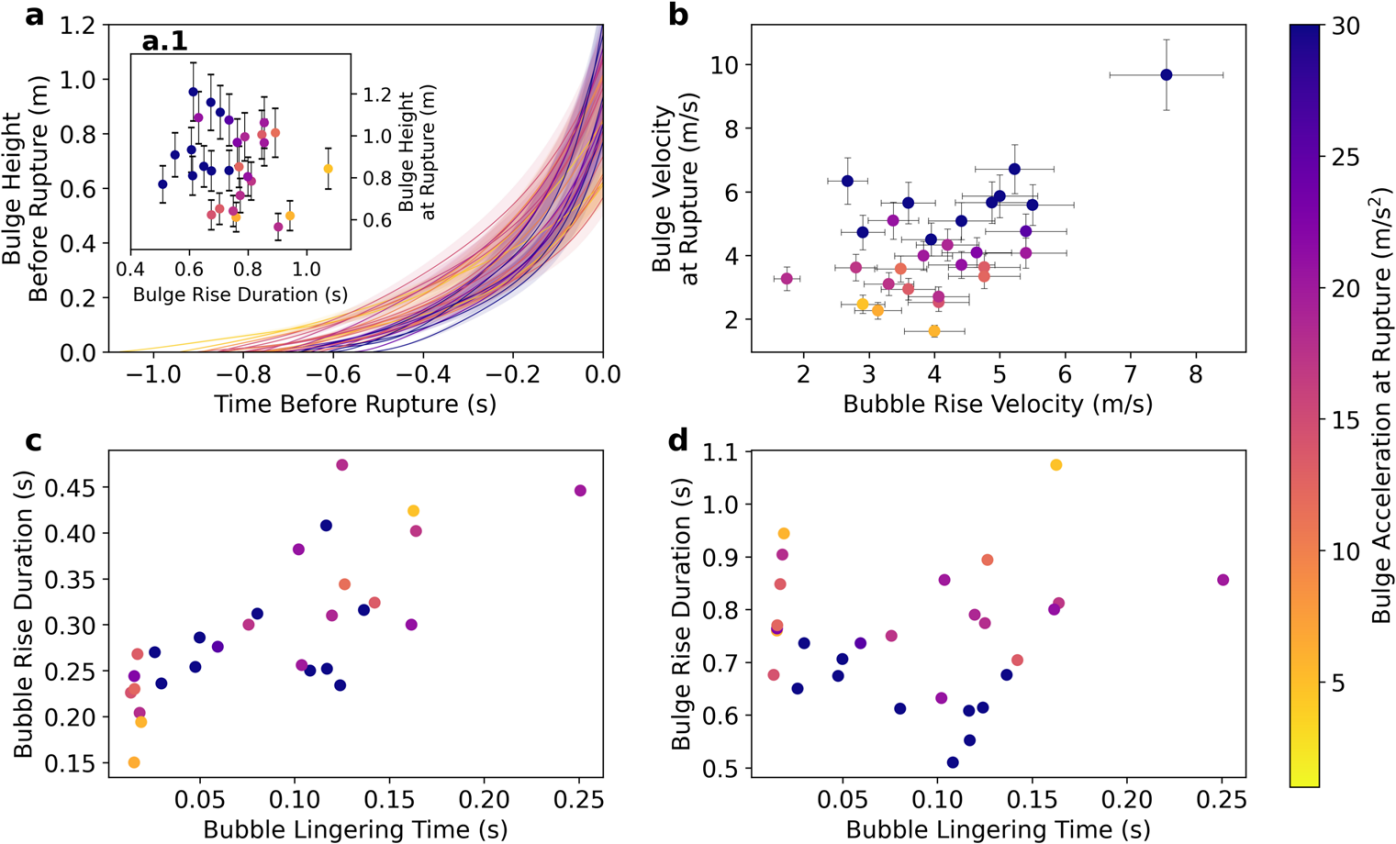
Bulge growth functions for 29 picked events aligned by the time of the rupture (*t*=0) (**a**). Bulge growth duration of each event is compared to the bulge height at the time of rupture (a.1). In addition, bubble rise velocity is compared to the bulge rise velocity at time of rupture onset (**b**). The video scaling uncertainty is represented as filled background areas (**a**) and error bars (a.1, **b**). Bubble lingering times are compared to the rise duration of the bubble cluster counted from the time of appearance in the video (**c**), as well as to the rise duration of the bulge (**d**). The color corresponds to the maximum acceleration of each bulge, which is right before the bulge ruptures

**Fig. 7:**
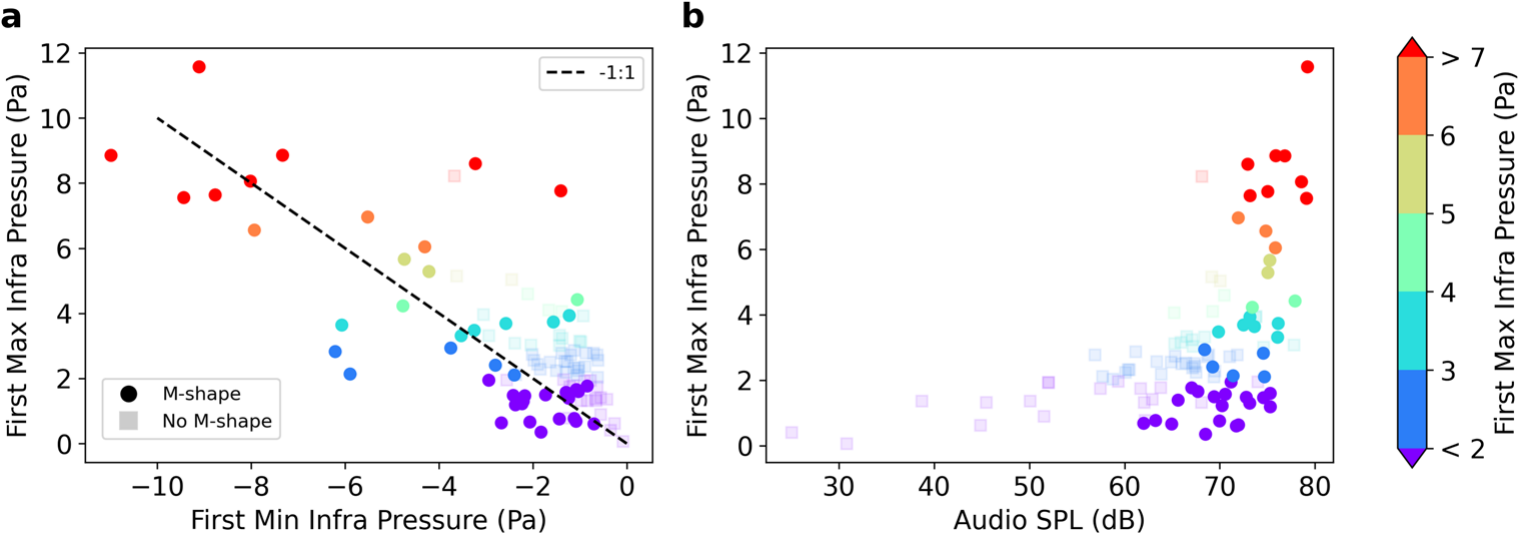
Relationship between acoustic amplitude parameters. **a** The amplitude of the first infrasound maximum (yellow triangles in Fig. 3a) and the following infrasound minimum (green triangles in Fig. 3a). The circle symbols show the result for the waveforms categorized as having the M-shape, whereas the squares show those that do not have an M-shape. The color shows the amplitude of the first infrasound maximum (same as the *y*-axis). Additionally the “1:1” relationship is shown as a black dashed line. **b** The relationship between the amplitude of the first infrasound maximum and the SPL audio amplitude. The symbols and colors are the same as in **a**

The approximated bulge height over time, derived from the kymograms (Fig. 6a), increases and accelerates up to the rupture point (Supplemental Figs. S11 and S12). The bulges showing the highest acceleration reach higher heights and rupture earlier (Fig. 6a.1). Comparing the bubble cluster dynamics to the bulge growth, a positive correlation is seen between the bubble rise velocity and the bulge rise velocity at the time of rupture (Fig. 6b). The bubble lingering time, which is the time delay between the bubble cluster reaching the top of the bulge and bursting, can last up to approximately 0.25 s. It is positively correlated with the bubble rise duration (Fig. 6c), which is measured from the moment the bubble cluster appears in the field of view. Overall, both shorter and longer lingering times are associated with the lowest final bulge accelerations and show longer bulge rise durations (Fig. 6d). The comparison between Fig. 6c and d shows that the bulge rise duration and bubble rise duration are not well correlated.

**Fig. 8:**
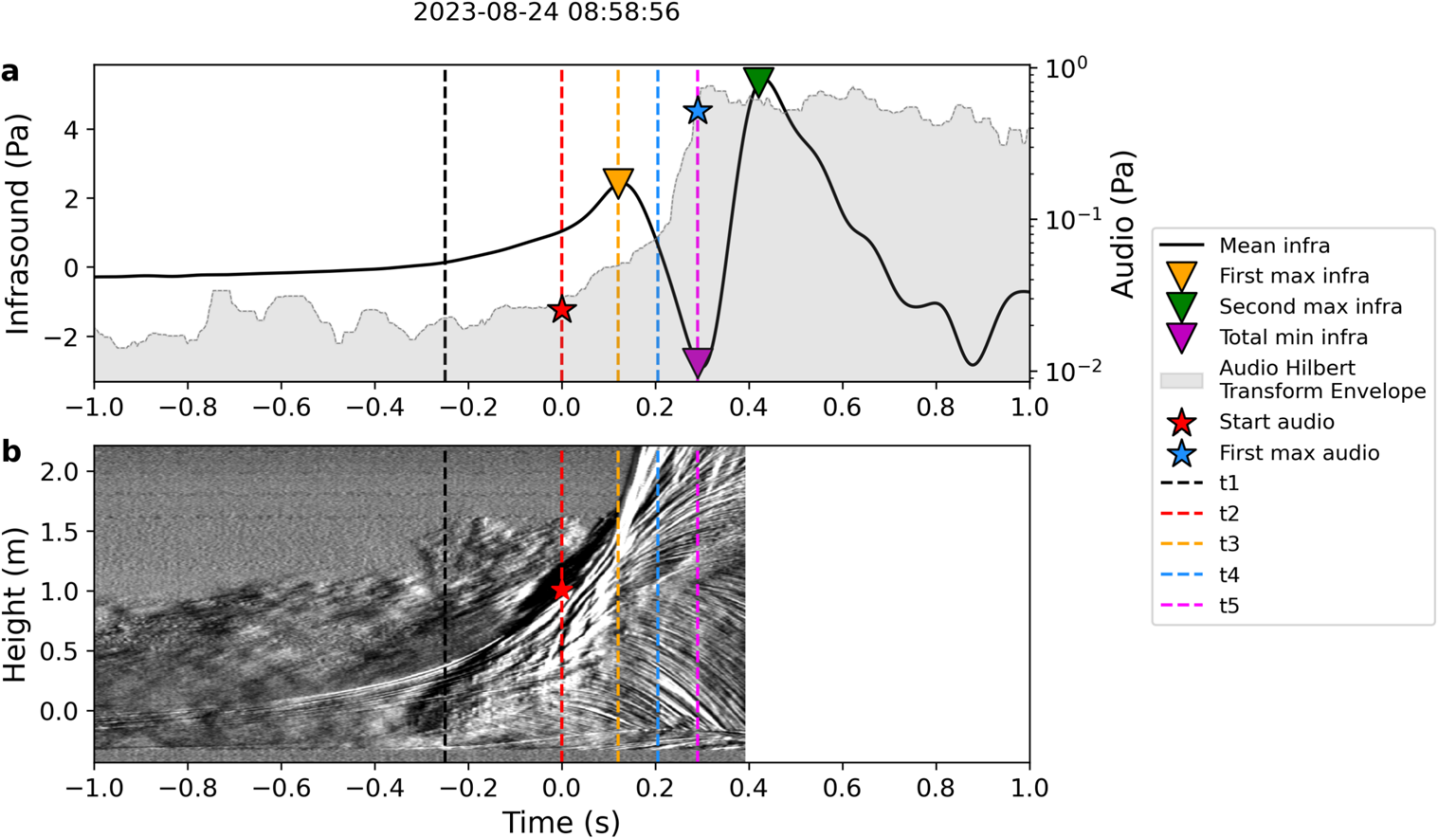
**a** Infrasound waveform and audio Hilbert transform envelope with symbols marking the acoustic parameters as in Fig. 3a. **b** Kymogram time-aligned with the acoustics in **a**. Both **a** and **b** show vertical lines labeled t1–t5, which are time snapshots corresponding to the video frames shown in Fig. 2 labeled t1–t5

### Acoustics

When filtering in the infrasound band, the amplitude is generally higher than in the audio band for the main events (Supplemental Fig. S1 compared to Fig. 1). When filtering in the audio band, amplitude peaks are visible between the main events (Supplemental Fig. S2). These correspond most likely to subsurface bubble collapses, as interpreted by seismic signals reported by Eibl et al. (2021).

We identify and show all 45 M-shaped waveforms filtered between 0.1 and 10 Hz and aligned by their first maximum in Fig. 3a. Waveforms that do not show an M-shape are shown in Supplemental Fig. S13. The M-shape is characterized by two pronounced infrasound maxima and centered minimum. After the second maximum, the waveform does not follow a general pattern other than tapering back to background over the next 1–2 s. On average, the time difference between the first and second infrasound maximum is 0.33 s with the minimum being at equal distance between the two maxima at approximately 0.16 s after the first maximum.

Comparing the timing of the low-frequency infrasound pattern and the high-frequency audio amplitude (Fig. 3a and b), we observe that the audio signal starts to increase on average 0.1 s before the infrasound peaks for the first time. The local minimum of the infrasound signal happens almost simultaneously with the maximum audio amplitude. The audio signal shows a time difference of approximately 0.29 s between its initial start and its first significant amplitude increase. The amplitude stays at an elevated level for approximately 1.5 s, which is longer than the infrasound signal. For both the infrasound and audio waveforms, the picked parameters have very similar timing between events, which points to a repeating source mechanism.

In addition to the relative timing, we also compared the relative amplitudes of the parameters picked above to help determine the acoustic source. Figure 7a shows that the amplitude of the first infrasound maximum and minimum generally follows a clear negative linear relationship and that they have the same absolute amplitude on average. This implies that both the compression and rarefaction are related to linear wave propagation or that the dynamics generating the compression and rarefaction obey the law of conservation of mass. Figure 7b shows that the first infrasound maximum is also positively correlated with the SPL audio amplitude. This means that the dynamics responsible for the infrasound amplitude are coupled to the dynamics that generate the audio acoustics with the first maximum about 0.19 s later. For both comparisons (Fig. 7a and b), events without an M-shape show a similar trend to those with an M-shape but generally have lower amplitudes. This means that the relationship between infrasound maximum, minimum, and SPL audio amplitude is independent from their self-similarity between waveforms. The second infrasound maximum amplitude does not show any significant correlation with the first maximum and minimum (Supplemental Figs. S14a-b). Furthermore, the time differences between the infrasound and audio parameters do not seem to correlate with the amplitude of the first infrasound maximum (Supplemental Fig. S14c).

### Comparison between acoustics and video

By synchronizing the video with the acoustic signals, the main features of the audio and infrasound signals can be linked to three general stages of the bulge dynamics (Figs. 2 and 8):

1. The bulge forms and starts to rise (Figs. 2 and 8t1)
  $\rightarrow$ Low frequency sound (infrasound) rises in amplitude.
  $\rightarrow$ High frequency sound (audio) stays at background level.
2. The bulge starts to rupture upward (or slightly to the side) (Figs. 2 and 8 t2)
  $\rightarrow$ Generally, infrasound amplitudes continue to rise.
  $\rightarrow$ Audio shows a sudden increase in amplitude above background level.
3. The bulge disintegrates (fountain erupts from the top of the bulge and sides of the bulge fall down) (Figs. 2 and 8t3–t5)
  $\rightarrow$ Infrasound continues to increase until its maximum, and then decreases in amplitude, after which it increases again (M-shape).
  $\rightarrow$ Audio stays at a high amplitude.

**Fig. 9:**
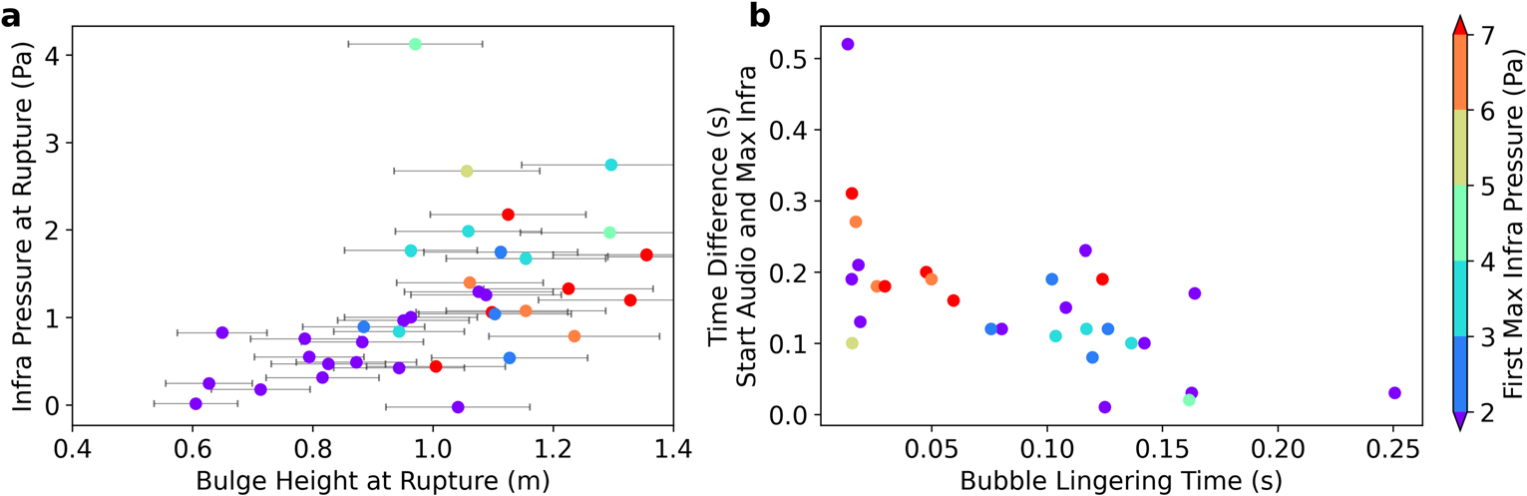
Scatter plots showing **a** the relationship between maximum bulge height and the infrasound pressure when the audio starts (proxy for bulge rupture) and **b** bubble lingering time versus time difference between the start of the audio and the first infrasound maximum. Colors are the same as in Fig. 7

We compare infrasound and audio parameters (derived in Fig. 3) with the bulge height at the start of the bulge rupture. The bulge height at rupture correlates positively with the infrasound pressure at the time of rupture as well as the maximum infrasound pressure (Fig. 9a and Supplemental Fig. S15a). This was also observed experimentally for bubbles ascending in pipe geometries (e.g., Lane et al. 2013). Similarly, we also see a positive correlation of both the infrasound maximum and the audio SPL with the bulge height at rupture (Supplemental Fig. S15b). The comparison between video and acoustic data shows a negative correlation between the bubble lingering time and the time difference between the start of the audio signal and the first infrasound maximum. In other words, shorter bubble lingering times are associated with larger time differences between the acoustic parameters (Fig. 9b).

**Fig. 10:**
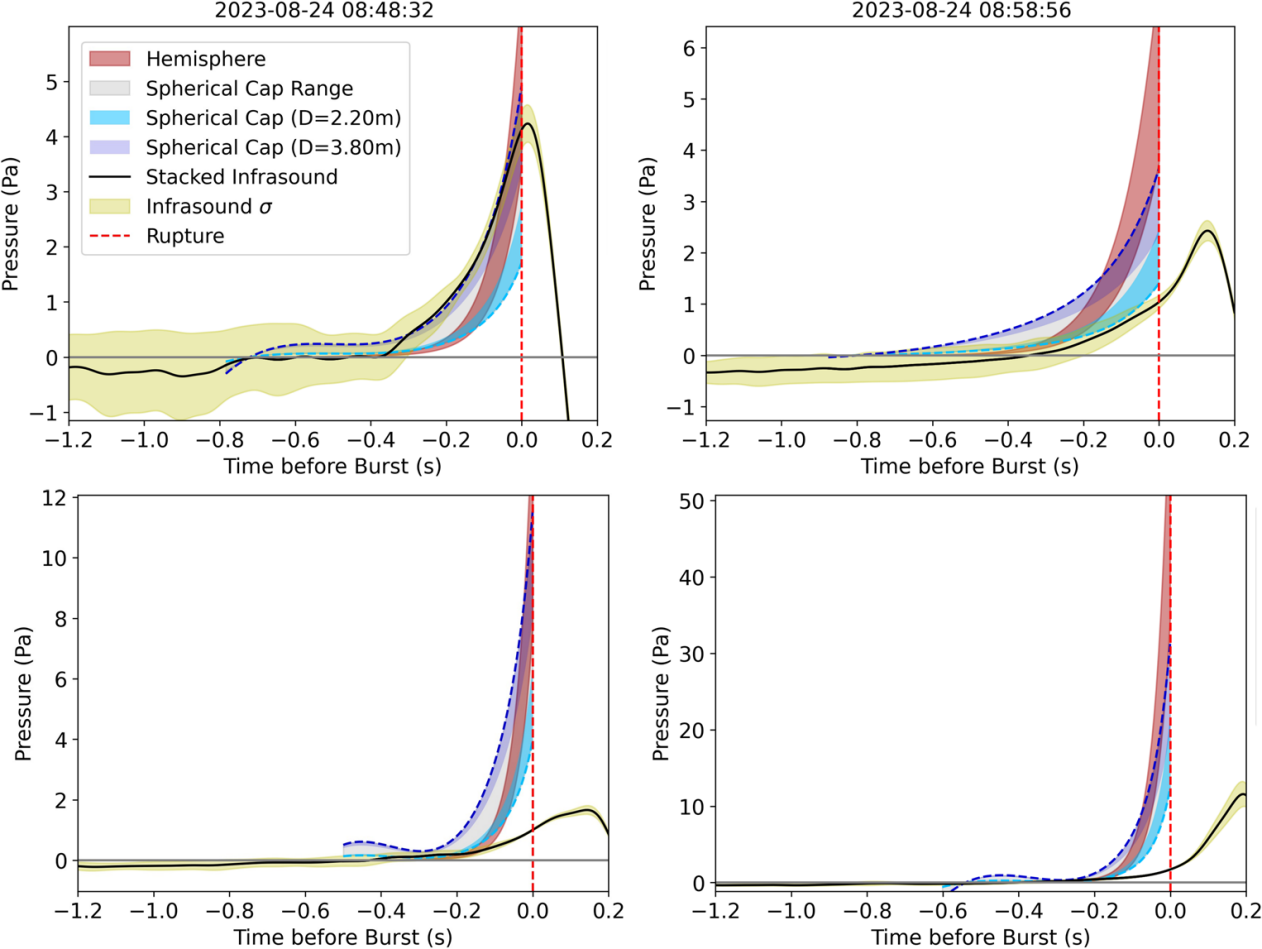
Example events showing the comparison between the recorded stacked infrasound (black) and its standard deviation $\sigma$ (yellow area) and the modeled acoustic pressure using the monopole model with the hemisphere approximation (red) and the spherical cap approximation with minimum (light blue) and maximum (dark blue) diameter. The range in area shows the variation in the result with different video scaling factors. The end of the modeled time series coincides with the start of the rupture (*t*=0). The associated bulge geometries and kymograms are shown in Supplemental Fig. S19

The pressure generated by the growth of the 29 bulges (Fig. 6) is modeled using Eq. 2 for the two different geometries: hemisphere (3) and the spherical cap (4). Furthermore, we use two different bulge diameters as extreme scenarios. We use the conduit diameter of *D* = 2.2 m as a minimum bulge diameter and maximum diameter reported by Eibl et al. (2024) of *D* = 3.8 m. As in the previous section, we take into account the range of scaling factors.

Qualitatively, the geometry of the spherical cap with the largest diameter of 3.8 m fits most bulges the best. Especially on the left (East) facing side, the geometry follows closely the shape of the bulge whereas the right (West) facing side shows a wider diameter than the modeled one (Fig. 4 and Supplemental Fig. S19).

**Fig. 11:**
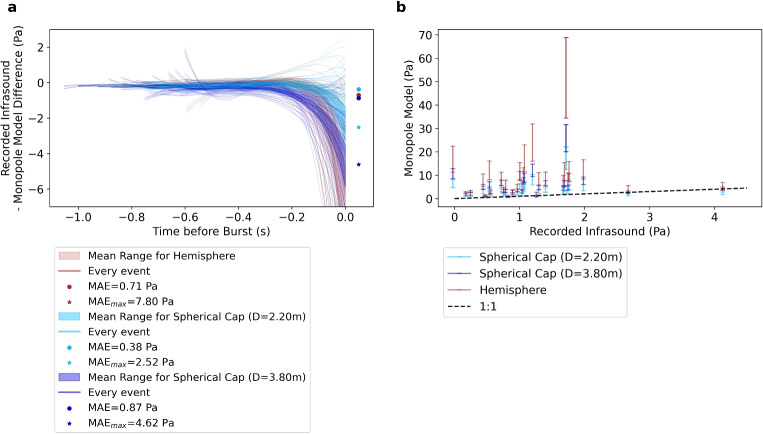
Difference between recorded infrasound and the monopole model using the volume of a hemisphere (red) and spherical cap with the minimum diameter of *D*=2.2 m (light blue) and the maximum diameter of *D*=3.8 m (dark blue). The filled areas show the average difference for each geometry using the range of video scaling factors. In **a**, the difference between recorded (stacked infrasound) and modeled pressure for each bulge is aligned at *t*=0. The circles show the MAE value using the entire bulge growth time series, and the stars show the MAE$_{max}$ value using only the time of the rupture (*t*=0). In **b**, the final pressures before the rupture of the bulge calculated using the monopole model are compared to the recorded infrasound. The error bars indicate the range of video scaling. The dashed black line shows where the modeled and recorded infrasound would coincide

Figure 10 shows the fit between the recorded infrasound data and the monopole model solution for four example events. First, the synthetic pressure curve starts at 0 Pa and accelerates in growth. In Fig. 10a and b, the observed infrasound pressure (black lines) lies within the range of modeled amplitudes. Specifically, in Fig. 10a, the recorded amplitude coincides best with the spherical cap approximation using the large diameter (dark blue), while in Fig. 10b, the recorded amplitude coincides best with the small spherical cap diameter (light blue). In Fig. 10c and d, the observed pressure is below the range of synthetic pressures. Figure 10c shows the deviation only right before the rupture (at $-$0.05 s), whereas Fig. 10d deviates even earlier with a very large pressure difference of >7 Pa at the time of the rupture.

To quantitatively evaluate the fit of all events, we calculated the difference between the recorded infrasound and the modeled synthetic pressure using the monopole model. Figure 11a shows the difference between the modeled and real waveform for the entire bulge growth. Generally, the difference is very low (<0.5 Pa) for the beginning of the bulge growth and deviates more strongly at the time of the rupture with a difference of more than 6 Pa in some cases. Most of the deviation is negative, meaning that the model overpredicts the recorded infrasound amplitude, which is also evident from Fig. 11b. We quantify the overall deviation between recorded and modeled waveforms by calculating the mean-absolute-error (MAE) over all stations (*n*) and all time steps (*t*): $\textrm{MAE}=1/(nt)\sum _n\sum _t|p(t,n)-m(t,n)|$. Furthermore, to evaluate the deviation at the time of the rupture (*t* = 0), we calculate the MAE$_{max}$ for the last time step only. We see the highest MAE and MAE$_{max}$ values for the hemisphere model with 0.73 Pa and 8.5 Pa. For the spherical cap approximation the MAE is between 0.35 and 0.90 Pa with minimum and maximum diameter, respectively. When only taking into account the time of rupture (*t* = 0), the MAE$_{max}$ value increases to 2.43–4.86 Pa.

The timing of rupture inception coincides with the rise in audio amplitude. As shown in Figs. 3 and 8, the timing of the rise in audio amplitude does not always coincide with the maximum infrasound amplitude. As explained in “Video,” the bulge appears to continue growing even after rupture has started, with the rupture area increasing over time. At some point, the water film of the bulge moves downwards while the water jet expands upward. This dynamic is visible as concave lines and marked in purple in the kymogram in Fig. 5. It is difficult to quantify the exact timing when the water film starts to collapse as it happens as a gradual process. Additionally, in Fig. 5, we observe that the timing of the apex of the concave lines does increase with height. This means that the collapse starts at the bottom of the bulge and moves upwards with time.

By comparing the infrasound waveform and the kymogram (Fig. 8), we observe that the apex of the concave disintegration patterns in the kymogram appears at lower heights around the time of the infrasound maximum. The disintegration then progresses upward between the infrasound maximum and the subsequent minimum.

**Fig. 12:**
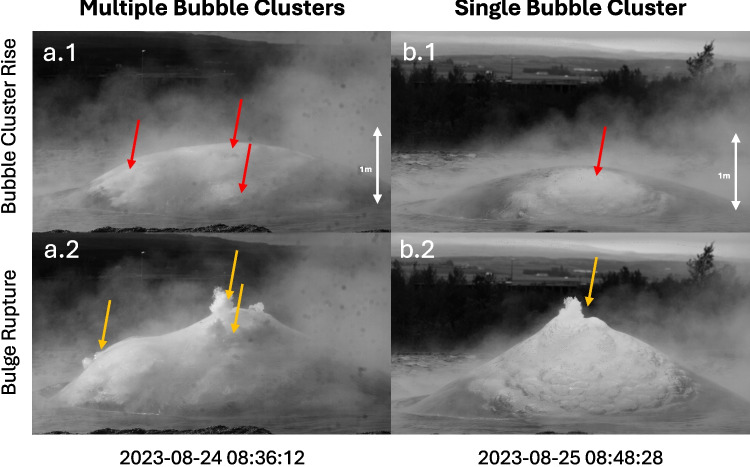
Video snapshots of the rise of two bulges, one with multiple bubble clusters (a) and one with a single bubble cluster (b). The frames before rupture (**a.1**, **b.1**) and during rupture (**a.2**, **b.2**) show red arrows pointing to the bubble clusters rising within the bulge and yellow arrows pointing to the rupture locations

## Interpretation and discussion

### Bulge growth

Bubble dynamics in a conduit (the flow of a slug or bubbly liquid in a pipe or a fissure) has been widely investigated both experimentally and theoretically (e.g., James et al. 2008; Lane et al. 2013; Del Bello et al. 2015; Capponi et al. 2017). As the bubble cluster rises, the bulge water film thins as the bulge grows and part of the water flows back down into the pool. The burst condition is met when the liquid film reaches a critical thickness and cannot withstand the pressure exerted by the bubble cluster at its top.

We found that the bulge growth duration is inversely correlated with its final height and acceleration (Fig. 6a and a.1). This means that the bulge height is not determined by a prolonged but rather a faster bulge growth, which is in agreement with experimental work. This is explained by a higher volume of expanding gas which leads to a greater expansion, a larger volume displacement and thus a faster and higher displacement of the liquid surface (James et al. 2008).

Our data show that the maximum bulge velocity correlates with the rise velocity of the bubble cluster (Fig. 6b), suggesting that the bulge growth is partially driven by the velocity at which the bubble cluster ascends to the surface. While the bubble lingering time correlates positively with the bubble cluster rise duration, it does not appear to correlate with the bulge rise duration (Fig. 6c and d). Physically, the bubble lingering time mirrors the balance between the upward buoyant force of the bubble cluster and the resisting tensile strength of the water film. Longer lingering times may indicate delayed rupture due to thicker or more stable water films, or due to clustering effects that distribute stress along the water/gas interface. Shorter lingering times may indicate more sudden ruptures, likely triggered by faster-rising clusters. Qualitative video analysis supports this interpretation. Events with long bubble lingering times, long bulge growth, and low accelerations are often associated with the rise of multiple bubble clusters (Fig. 12a.1). In these cases, rupture is initiated at multiple locations, either simultaneously or in rapid succession (Fig. 12a.2). This behavior may be related to splitting of single bubbles into multiple bubbles. This was observed experimentally for bubbles ascending through widening conduit geometries (James et al. 2006), a scenario similar to Strokkur, where the conduit exhibits multiple changes in cross-section and is widest near the top (Walter et al. 2020). In contrast, events with short bubble lingering times are associated with smaller and slower bulges, showing long growth durations and low acceleration. In these cases, a single bubble cluster appears comparatively late in the video sequence relative to the duration of the bulge rise (Fig. 6c). This could indicate that the bubble cluster has a relatively high velocity, leading to an anticipated piercing of the water film (short lingering time). Finally, events with intermediate bubble lingering times show larger bulges characterized by the highest acceleration, shortest growth duration, and a single bubble cluster (Fig. 12b). For further examples of bulges with multiple or single bubble clusters rising and rupturing, see Supplemental Figs. S20 and S21.

**Fig. 13:**
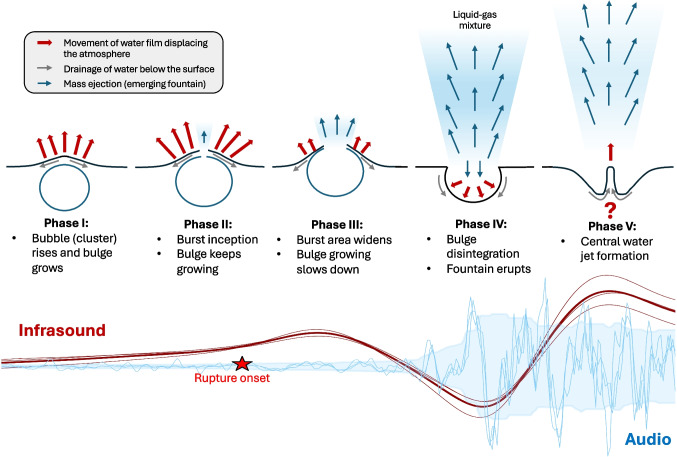
Conceptual model of the growth, rupture and disintegration of the geyser bulge. The red arrows highlight the direction of movement of the water surface, bulge, and bubble film. The blue arrows show the movement of the turbulent gas-water mixture that is expelled after the bulge ruptures. The time-aligned waveforms at the bottom show the associated infrasound (red) and audio (blue). The blue shaded background is the Hilbert transform of the averaged audio signal

### Acoustics of bulge growth

The low- and high-frequency acoustic waves show very different waveforms, which coincide with different scales such as bulge growth and jetting, as well as different phases of the bulge growth and rupture (Figs. 8 and 13). Generally, the acoustic amplitude positively correlates with bulge dynamics, like bulge height, velocity, and acceleration, which indicates that the source of the acoustic waves, both infrasound and audio, is directly related to the bulge growth. This is different to the seismic energy, which we did not investigate here, which seems to anti-correlate with the height of the bulge and also the fountain. This could be the result of differences in the seismic coupling between the rising bubbles and the conduit walls (Eibl et al. 2024). This further emphasizes the advantages of acoustic measurements as subsurface dynamics are directly coupled into the atmosphere. The following results and discussion primarily focus on comparing video and acoustic data from events that exhibit the characteristic M-shape, which are typically associated with higher acoustic amplitudes and greater bulge heights. Events with infrasound waveforms lacking a distinct pattern remain an avenue for future investigation. To distinguish events with characteristic waveform shapes, such as those exhibiting an M-shape, we tested clustering algorithms. However, manual classification provided the most consistent separation. Similarly, for extracting acoustic parameters, manual picking proved more reliable than automatic methods. Although some uncertainty exists (± 0.01 s), this has minimal influence on time-dependent parameters and does not affect the interpretation of key trends.

The infrasound signal rises distinctly during the initial bulge growth, very similar to the infrasound signal recorded and described at Erebus Volcano (Gerst et al. 2013). This is followed by the emergence of the high-frequency audio signal, which coincides with the onset of rupture. We interpret the continued rise of the infrasound waveform after the rupture onset as indicative of ongoing bulge growth while the rupture area gradually widens (phases I–III in Fig. 13).

Our monopole acoustic model assumes that the acceleration of the atmospheric displacement by the bulge produces the recorded pressure. Other variations of this model, such as the Rayleigh integral (e.g., Blom et al. 2020; Bowman and Krishnamoorthy 2021), are not applicable as they assume a piston-like volume displacement. Furthermore, integrating over the bulge surface with each surface area contributing as a monopole (similar to Gerst et al. (2013)) did not deviate much from the simple monopole source model proposed here. We therefore favor the simple monopole model due to its lower complexity and as a base to build upon in future studies. The range of modeled amplitudes for each event is most likely due to uncertainties in the geometry of the bulge, such as bulge diameters and video scaling (Figs. 10 and 11). Generally, the modeled amplitudes fit the recorded amplitudes reasonably well for the spherical cap volume approximation with a small diameter. Interestingly, the actual diameter of the bulge is more closely modeled by the larger diameter which over-predicts the recorded amplitude in most cases. The differences between the model and recordings are possible due to a variety of factors. Using the monopole model, we assume a uniform expansion of the bulge. However, as explained in “Acoustics,” the bulge is better approximated by a spherical cap with mostly constant diameter and changing height. Therefore, the top of the bulge is displacing more volume than the sides which could lead to an unequal distribution of pressure around the bulge. As the infrasound sensors are placed on the ground, they could be subjected to a lower pressure. Furthermore, the bulge is not symmetrical and could show a different rate of expansion orthogonal to the camera’s view which is not visible in the videos used in this study. Most videos show a larger radius to the right (West) facing side of the camera’s view (Fig. 4 and Supplemental Fig. S19). This asymmetry is not reflected in a consistent increased pressure towards this side (Supplemental Fig. S19). Picking the bulge heights and approximating it with a 5th degree polynomial yielded the most reliable approximation, as edge-detector methods and other automated techniques failed to accurately capture the bulge height. However, remaining inaccuracies lead to errors when taking the second derivative for the volume acceleration. This is also the reason for the partially wavy synthetics as shown in Fig. 10d. Furthermore, there could be inaccuracies in picking the start time of the bulge rupture as this happens gradually and is often not centered on top of the bulge. Moreover, the used model is a simplification of the natural process and might need to be adapted for the close distance between the sensors and the geyser and the geometrical expansion in all directions. Future measurements of the 3D-wavefield could help the interpretation of the acoustic source in terms of directionality and appropriateness of a multipole model approach (e.g., Iezzi et al. 2019, 2023).

### Bulge rupture and disintegration

As shown in Figs. 8 and 13, the bulge rupture coincides with the start of elevated audio signals. The burst releases pressure rapidly, which can be compared to small scale jetting where the frequency content scales with the jet diameter (Gestrich et al. 2022). The rupture gradually widens and is mirrored in the slow increase in audio amplitude. In most cases, this slow increase in amplitude is followed by a sudden increase, which could be associated with either the sudden widening and energetic disintegration of the bulge or by additional jet-like features associated with the emerging of the water fountain.

The initial infrasound increase is interpreted to be generated by the acceleration of the atmospheric displacement by the growth of the bulge. The subsequent maximum and following decrease of the infrasound amplitude could therefore be related to a decrease of the bulge acceleration as the gas escapes it (see phase III in Fig. 13). The time difference between the start of the audio signal and the infrasound maximum can thus be interpreted as the time it takes for the rupture to release enough pressure for the bulge to disintegrate, henceforth called rupture duration. This time difference correlates negatively with the bubble lingering time (Fig. 9b), i.e., shorter bubble lingering times correspond to an overall longer rupture duration. Based on the interpretation in “Bulge growth”, the shorter bubble lingering times correlate with small, slow-rising bulges. In these cases, the rising bubble cluster initiates the rupture process early, before significant pressure buildup. This leads to a prolonged rupture duration, as evidenced by the long delay between the initial audio signal and the first infrasound maximum. Conversely, the longer bubble lingering times are associated with more than one bubble cluster rupturing the bulge surface at multiple points (Fig. 12a) resulting in a shorter rupture duration. This observation highlights the potential for inferring bubble cluster dynamics by comparing acoustic parameters across different frequency bands and for different systems as gradual rupture processes have been observed at lava and mud volcanoes (Rudolph et al. 2024).

During the rupture, while most of the bulge liquid mass is ejected upwards, some moves downwards (purple lines in Fig. 5) which could be caused by the decompression of the bubble cluster and the subsequent inwards movement of the bubble film (phase IV in Fig. 13). Such velocity reversal has been modeled before for bubbles bursting at a water-air interface (e.g., Boulton-Stone and Blake 2006; Deike et al. 2018). The observed downward movement in the kymogram (Fig. 5) is originating near the pool surface and propagating through the bulge over approximately 0.15 s. The duration of the downward movement varies between events and it is difficult to be precisely determined, as it may be superimposed by the collapse of the water fountain. However, the downward movement consistently begins between the first infrasound maximum and its subsequent minimum, as shown in Fig. 8 and the videos in the Supplemental Material.

The M-shaped infrasound waveforms have two clear maxima. This shape has been observed before in geophysical data related to similar natural or artificial phenomena. Vergniolle and Brandeis (1996) related the waveform observed during Strombolian eruptions to vibrations or fluctuations of the bulge, which forms when the gas slug rises and pushes up the magma, forming a thin film until it ruptures. This model has been used to calculate eruption dynamics, such as during the primarily submarine eruption of Bogoslof (Lyons et al. 2019). The phenomenon of the fluctuating bulge radius has been observed visually by Bouche et al. (2010) at the Erta Ale lava lake in Ethiopia. The field observations and high-speed videos of Strokkur geyser provided a very clear view on the dynamics of the rising bulge and did not show any oscillations of the bulge radius. Therefore, it is unlikely that this is the cause for the M-shape. Gerst et al. (2013) explains the second infrasound peak and those that follow by cavity resonance in the entire length of the gas-filled slug that previously ruptured at the top. At Strokkur geyser, the fountain eruption is driven by a cluster of bubbles rather than a slug, making resonance an implausible cause for the observed M-shape.

During shallow chemical underground explosions, the M-shape is explained by an initial acceleration of the ground due to the explosion itself, which leads to a spallation and subsequent downfall of the ground, generating the decompression part of the waveform. The following rebound, called “spall closure” is responsible for the second compressive peak (e.g., Blom et al. 2020; Bowman and Krishnamoorthy 2021). Another possible explanation is a standing wave in the pool of Strokkur, also called a seiche. However, using $\tau =\frac{2L}{\sqrt{gH}}$ by Rueda and Schladow (2002) with the diameter of the pool L$\approx$8 m and depth H$\approx$0.1–0.5 m, we calculate a period of $\tau \approx$ 7–16 s, which is much longer than the measured 0.32 s between the two maxima. Ichihara et al. (2009) observed a similar pattern, also referred to as an M-shape, from an underwater explosion, which produces fountain-like subsurface dynamics. They hypothesized that this shape is generated by a “sultan”, which is a center acceleration, closely resembling a fountain after the initial acceleration of the water surface due to the rising bubble. However, the duration between the first and second peak of their recorded M-shape waveform is ca. 0.02 s, whereas the one measured at Strokkur is generally about 16 times as long. Other forms of jetting after bubble burst have been observed experimentally and numerically (e.g., Boulton-Stone and Blake 2006; Deike et al. 2018; Li et al. 2019). They explain that the formation of the jet is caused by the water film falling back down after rupture and collecting at the bottom. The inward force of the water subsequently generates an upwards directed jet (phase V in Fig. 13). The timing between the burst of the bubble and the emergence of a jet are difficult to quantify and needs more research. However, we believe that this could be a viable explanation of the second infrasound maximum as we exclude the possibility of resonance, oscillations, or the formation of a seiche. Further investigation of this phenomenon will need to be conducted to explore the possibility of this dynamic in a geyser setting as well as for volcanoes.

### Implications for volcanoes

Geyser systems share many similarities with volcanoes, particularly in terms of subaerial dynamics and air displacements, which are common sources of acoustic signals. Here, we focused on the growth and rupture of the water bulge, considering it broadly analogous to the degassing dynamics observed in open-conduit, low-viscosity volcanic systems. Overpressured ascending bubbles and gas-filled slugs are common at Strombolian (Wilson and Head 1981; Jaupart and Vergniolle 1989; Vergniolle and Brandeis 1996) and Hawaiian eruptions, including lava lake dynamics at Kilauea (Hawaii) (Mintz et al. 2015), Erebus (Antartica) (Gerst et al. 2013) and Erta Ale (Ethiopia) (Bouche et al. 2010), and mud volcanoes (Rudolph et al. 2024). The subaerial dynamics are very similar to what is observed at Strokkur geyser. Reported bulge growth velocities for lava bulges at Kilauea volcano range from 4–16 m/s. Though slightly higher, this is similar to the final bulge rise velocities observed at Strokkur, ranging from 2–8 m/s (Fig. S12a). Likewise, lava bubble heights of 1–5 m at Kilauea are somewhat greater than the 0.5–1 m bulge heights measured at Strokkur (Fig. 6a). In contrast, bulges at Erebus volcano are significantly larger in scale, with estimated heights often exceeding 5–10 m and rise velocities up to 60 m/s before rupture. Vergniolle and Brandeis (1996) estimated bulge radii of 0.6–1.2 m at Stromboli volcano, comparable to those observed at Strokkur, but reported significantly higher maximum radial velocities of 10–60 m/s. Opposite to geysers, conduit conditions and explosion dynamics at active volcanoes change more rapidly, leading to greater variability in bulge velocities and radii. Both latter examples use a variation of the monopole source model provided in Eq. 2 to model the bulge rise. Similarly, Yokoo et al. (2008) also used an infrasound model to simulate the initial swelling of a lava plug at Sakurajima volcano, Japan. However, in that case, the swelling occurred over a comparable duration (0.3 s) and ended in an abrupt explosion, marked by a sudden increase in infrasound pressure rather than a gradual rupture.

However, bulge dynamics following the initial swelling, such as oscillations (e.g., Vergniolle and Brandeis 1996; Bouche et al. 2010), and resonances (Gerst et al. 2013), are not observed during the bulge rise and rupture at Strokkur. We therefore hypothesize that this difference arises from contrasts in viscosity, thermal conditions, and resulting force balance, which causes the dynamics of a rising and rupturing water bulge to differ from those of a lava bulge. Future investigations could explore whether the central jet generated by the down-falling magma film (phase V in Fig. 13) might explain the second infrasound peak.

## Conclusion

This study provides a comprehensive analysis of the acoustic and physical processes involved in the formation and rupturing of water bulges at Strokkur geyser, with a focus on the first two seconds of the bulge rise and subsequent eruption. Infrasound sensors and high-speed video recordings were used to track the dynamics of the bulge and correlate the acoustic signals with visual data. Distinct acoustic signatures, including a notable M-shaped infrasound waveform, were regularly identified. We correlated different portions of the water bulge rise and rupture with the various portions of the M-shaped waveform. Further, we successfully applied a monopole model to simulate the pressure variations associated with the expanding bulge. Our modeled amplitudes and waveforms are generally consistent with the infrasound data recorded at Strokkur.

Through synchronized acoustic and video measurements, we demonstrated that infrasound can effectively detect the growth of the bulge, even before the visual eruption occurs, and audio sound can detect the onset of the bulge rupture. The analysis revealed that the timing and amplitude of the acoustic signals provide critical insights into the bulge dynamics, from its formation to its rupture and disintegration. The comparison of the acoustic and visual data highlighted the progressive nature of the bulge rupturing and the complexities of its disintegration. In particular, we found that the timing of acoustic parameters across multiple frequency bands correlates with bubble cluster dynamics. These findings underscore the value of acoustic measurements as a powerful tool for investigating subsurface volcanic and geyser processes, offering a non-invasive method to understand the dynamics driving eruptive phenomena.

Our findings can be extended into volcanic research and monitoring, as the processes governing geyser eruptions share similarities with those in volcanic systems, particularly in terms of fluid and gas dynamics. Specifically, characterizing the time, size, velocity, and acceleration of magma bulges could be used for the near real-time analysis of eruption dynamics and characterization of changes in the system. Strokkur geyser, with its regular and relatively safe eruptions, serves as a useful analogue for understanding larger and more dangerous volcanic events. This research contributes to improving the interpretation of geophysical signals in both geyser and volcanic environments, providing a framework for future studies on eruption dynamics.

## Supplementary Information

Below is the link to the electronic supplementary material.

Supplementary file 1 (mp4 45786 KB)

Supplementary file 2 (pdf 23574 KB)

## Data Availability

The high-speed video recordings and acoustic data used in this study are openly available through GFZ Data Services. The video dataset is available at https://doi.org/10.5880/fidgeo.2025.068 (Gestrich et al. 2025a), and the acoustic dataset is available at https://doi.org/10.5880/fidgeo.2025.069 (Gestrich et al. 2025b).
