# Supplementary material for: The acoustics of bulge rise and rupture at Strokkur geyser
Source: Bull Volcanol. 2025 Sep 13;87(10):82. doi: 10.1007/s00445-025-01876-3 (PMC12433364; doi:10.1007/s00445-025-01876-3)
Supplement: Supplementary file 2 — (pdf 23574 KB) [file 445_2025_1876_MOESM2_ESM.pdf]

Supplemental Material

The Acoustics of Bulge Rise and Rupture at Strokkur Geyser

Julia E. Gestrich[1*], Corrado Cimarelli[1], David Fee[2], Antonio Capponi[1], Caron Vossen[1], Markus Schmid[1]

[1*]Department of Earth and Environmental Sciences, Ludwig-Maximilians Universität München, Theresienstraße 41, München, 80333, Germany.
[2]Geophysical Institute, University of Alaska Fairbanks, 2156 Koyukuk Dr, Fairbanks, 99775, AK, USA.

*Corresponding author(s). E-mail(s): julia.gestrich@min.uni-muenchen.de;
Contributing authors: cimarelli@min.uni-muenchen.de; dfee1@alaska.edu; antonio.capponi@min.uni-muenchen.de; caron.vossen@min.uni-muenchen.de; markus.schmid@min.uni-muenchen.de;

1 Supplemental Figures for Section “Acoustics”

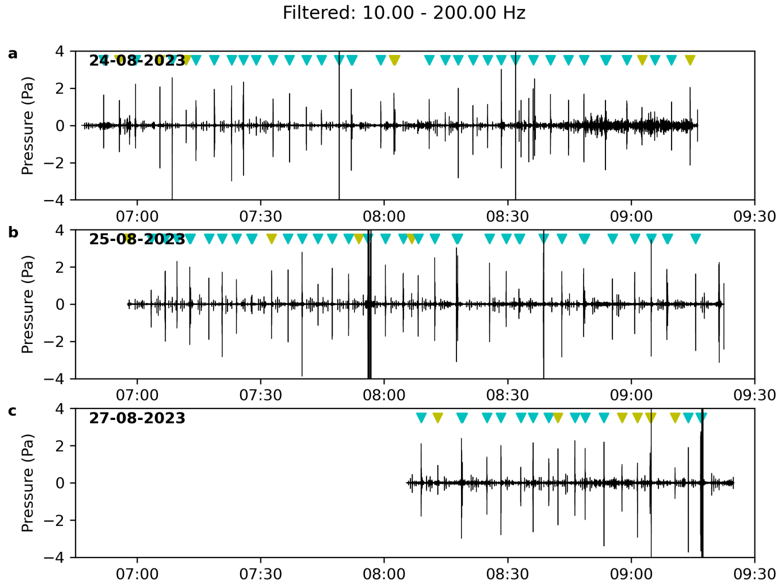

Figure S1 Overview of the acoustic waveforms recorded between August 24 - 27 2023 (a)-(c). The black line shows the waveform filtered in the high frequency (audio) band between 10 - 200 Hz. The triangles above the waveform mark the timing of the fountaining events and whether they were captured by video (blue) or not (yellow).

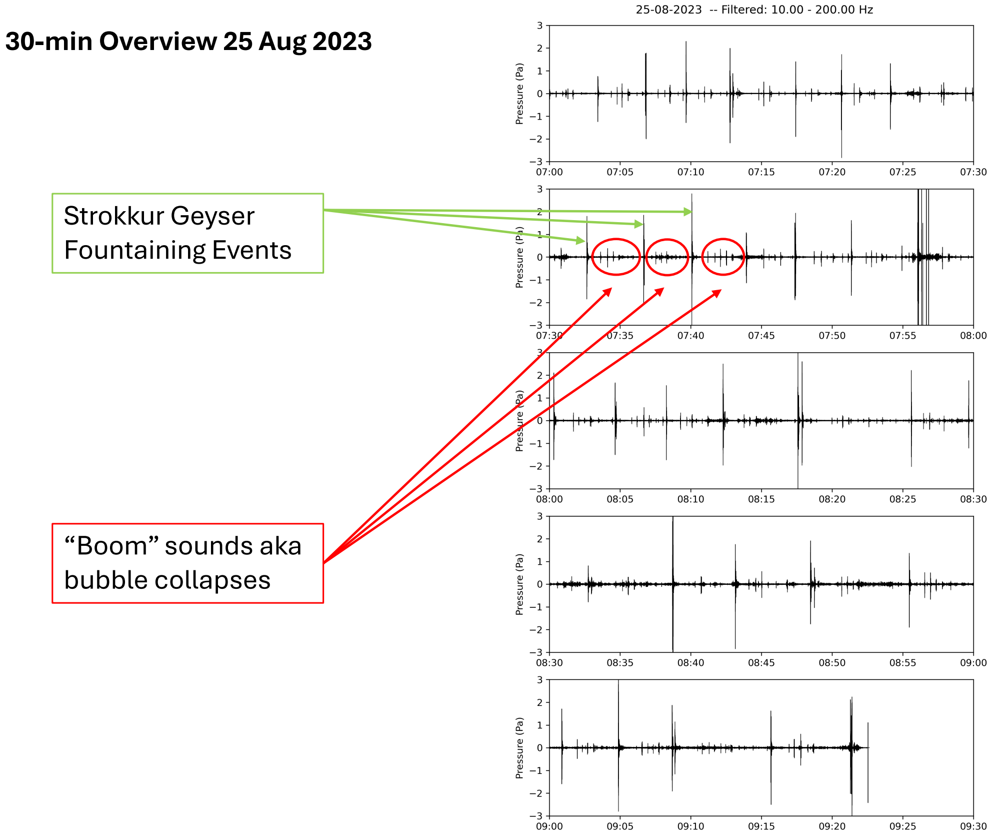

Figure S2 Overview of high frequency audio data for August 25 with annotated timings of fountaining events and bubble collapses at depth.

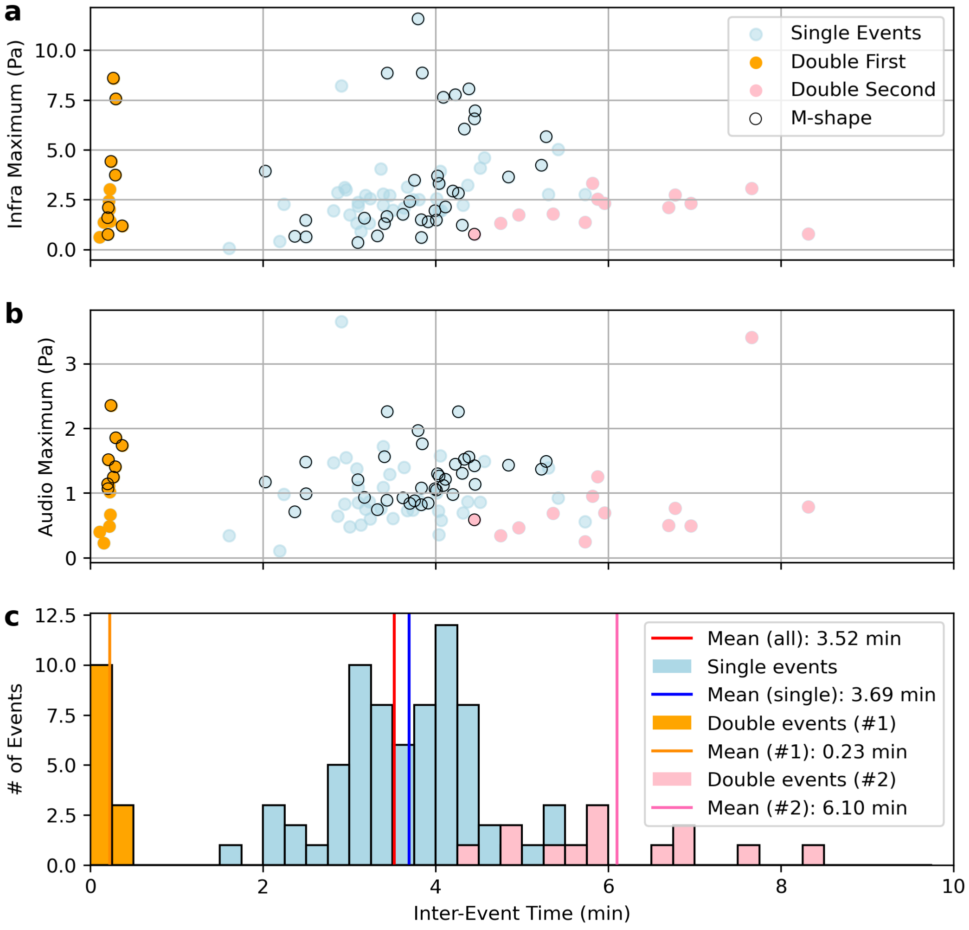

Figure S3 Inter-event times. (a) shows the maximum infrasound amplitudes versus the inter-event time afterwards. (b) shows the maximum audio amplitudes versus the inter-event time. The light blue circles are the single events while the orange colored circles are the first event of a double eruption and the pink colored circles are the corresponding second event. Circles with a black edge are events that are categorized as showing an “M”- shape. (c) shows a histogram of the number of events within the 0.25 s interval of the bars. The colors of the histograms denote the subset of events considered for the histogram and the vertical lines show their mean with blue corresponding to the single eruptions, orange to the first event of a double eruption and pink to the second event of a double eruption. The red vertical line is the mean inter-event time of all events.

2023-08-25T08:12

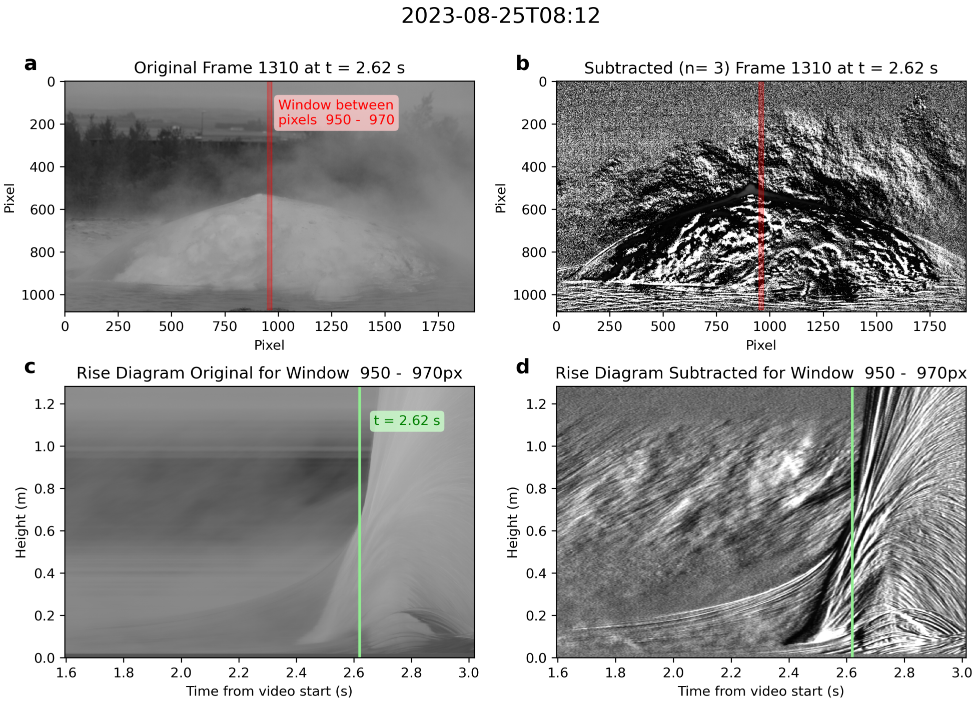

Figure S4 Video processing steps for the geyser event on August 25 at 8:10 UTC. (a) shows the original frame number 1248 (∼2.5 seconds after the start of the video). (b) shows the same frame as in (a) but with applied background subtraction of the third last frame. (c) shows the kymograph using the window marked in red in (a). The green line shows the timing of the frame shown in (a) within the kymograph. (d) shows the kymograph for the video with applied background subtraction.

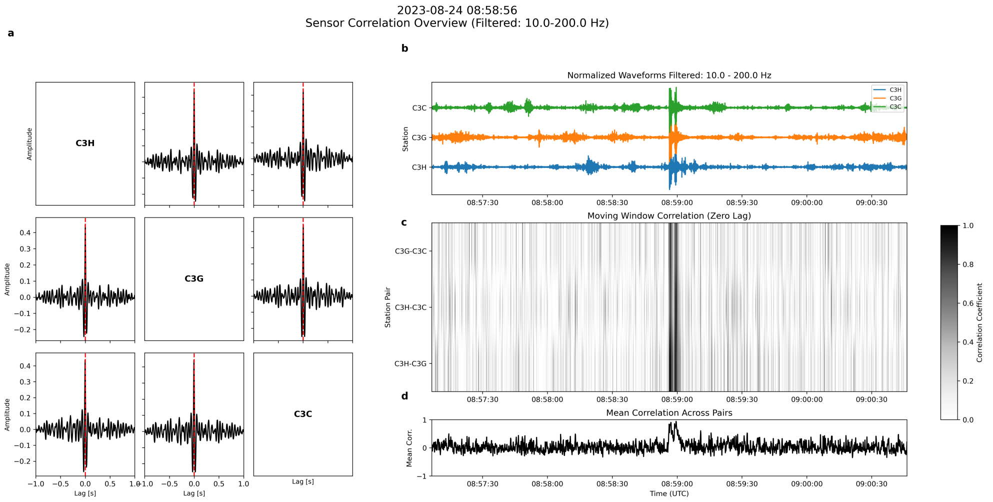

Figure S5 Correlation results between all active infrasound sensors for the event at 08:58:56 UTC (2023-08-24). For this example only three of the infrasound sensors were active at this time with signals filtered in the high frequency band (audio) between 10 – 200 Hz. (a) shows a matrix of cross correlation results between all sensors with the highest correlation at 0 s (red dashed line). (b) shows the filtered waveforms used for the analysis. (c) shows the Pearson's correlation coefficient between all sensors (combinations named on the y-axis) for a moving 1 s window with 90 %overlap. (d) shows the mean Pearson's correlation coefficient between all sensor pairs.

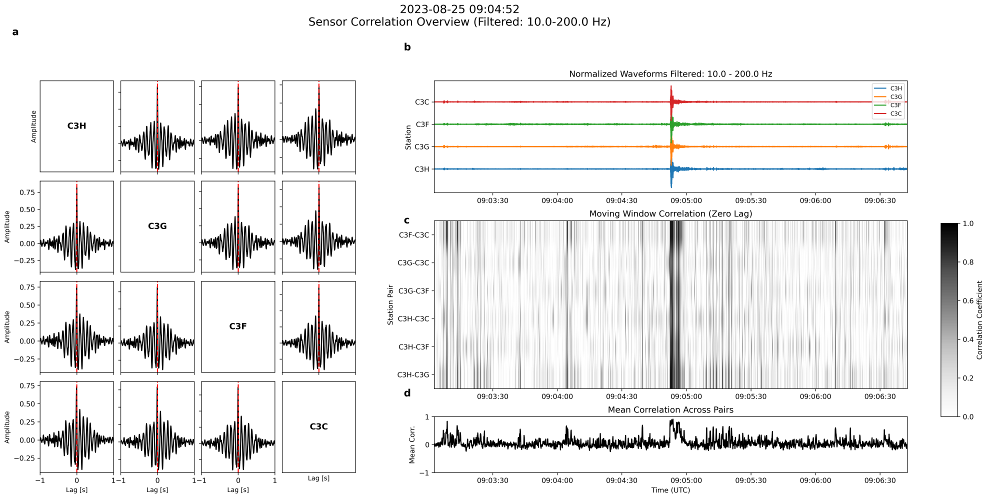

Figure S6 Correlation results between all active infrasound sensors for the event at 09:04:52 UTC (2023-08-25). For this example only three of the infrasound sensors were active at this time with signals filtered in the high frequency band (audio) between 10 – 200 Hz. See Figure S5 for description of the panels.

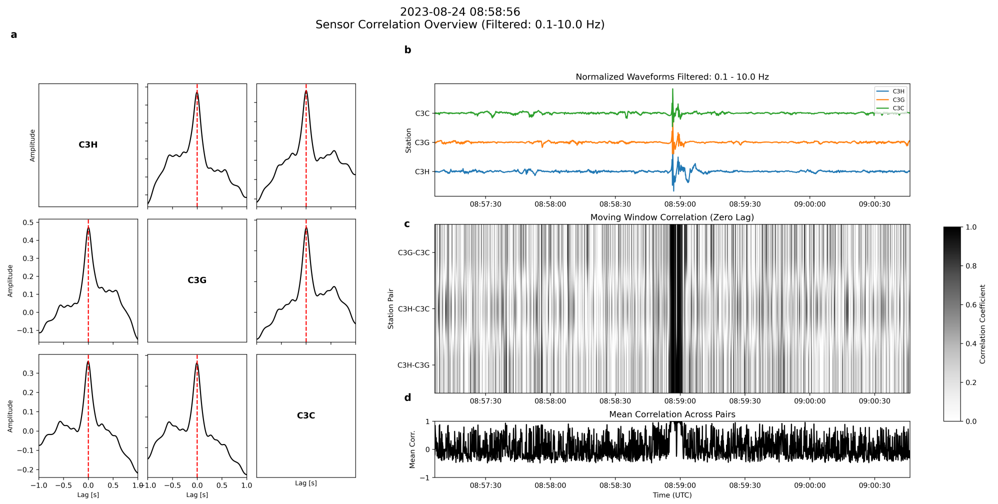

Figure S7 Correlation results between all active infrasound sensors for the event at 08:58:56 UTC (2023-08-24). For this example only three of the infrasound sensors were active at this time with signals filtered in the low frequency band (infrasound) between 0.1 – 10 Hz. See Figure S5 for description of the panels.

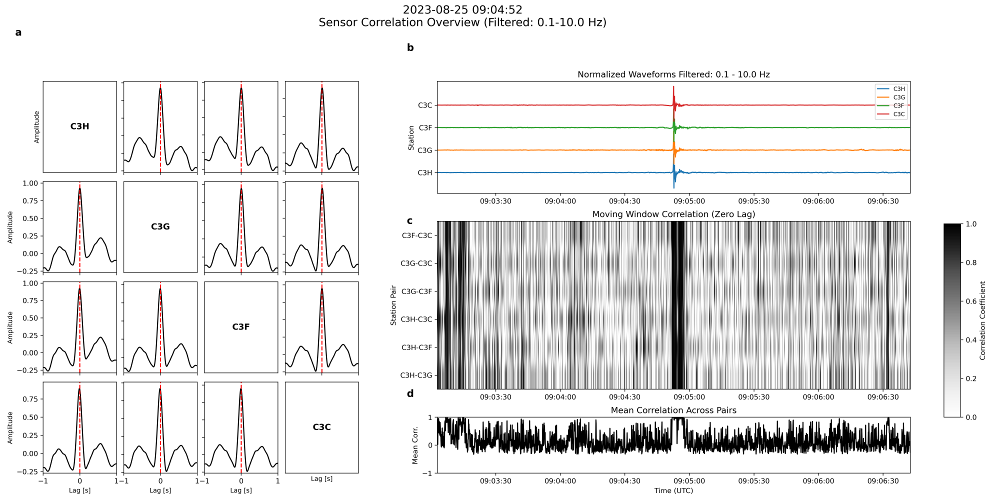

Figure S8 Correlation results between all active infrasound sensors for the event at 09:04:52 UTC (2023-08-25). For this example only three of the infrasound sensors were active at this time with signals filtered in the low frequency band (infrasound) between 0.1 – 10 Hz. See Figure S5 for description of the panels.

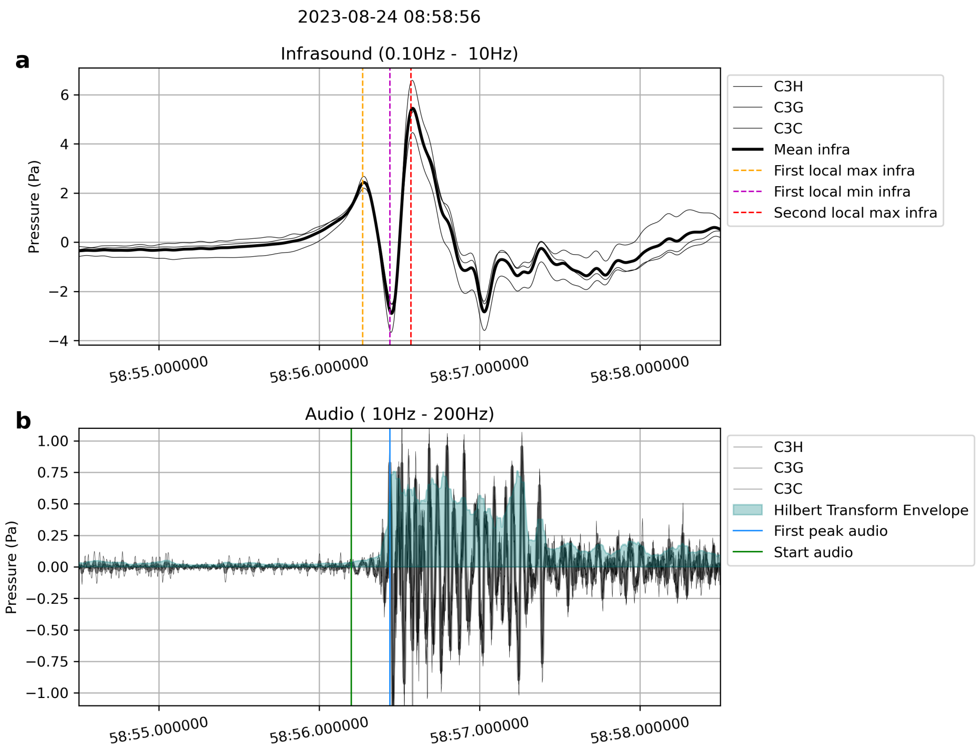

Figure S9 Example event (a) infrasound and (b) audio waveforms for each sensor (thin black lines) and stacked mean waveform (thick black line). The Hilbert transform envelope is shown as a blue area in (b). The timing of the picked acoustic parameters are shown as colored vertical lines.

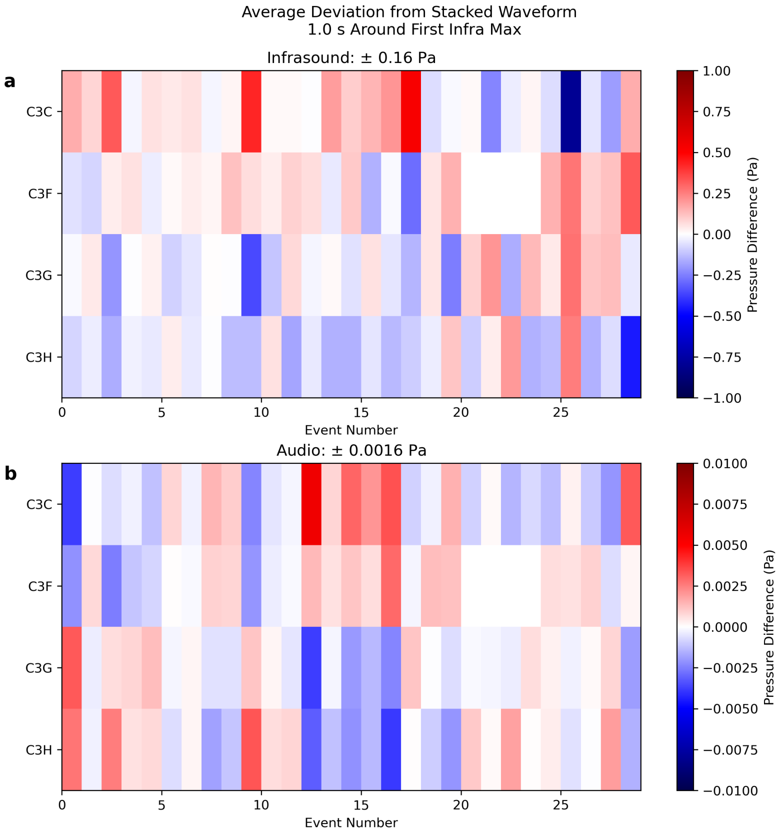

Figure S10 Amplitude variation of each sensor for each of the 29 analyzed events compared to the stacked waveform in a 1 s window around the first infrasound maximum. (a)shows the result for the signal filtered between $0.1-10$ Hz and (b) shows the result for the signal filtered between $10-200$ Hz. The deviations are very small with ± 0.16 Pa for the infrasound signal and ± 0.0016 Pa for the audio signal. Each sensors amplitude variation is sometimes positive and sometimes negative confirming that no sensor has a systematic higher or lower amplitude.

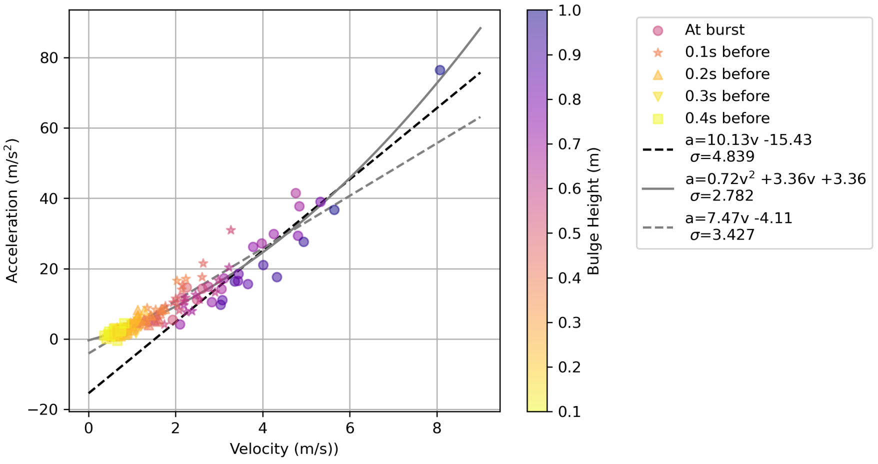

Figure S11 Relationship between bulge rise velocity and acceleration with time. The different symbols denote the timing relative to the time of the burst. The color of the symbols shows the bulge height at this time. The black dashed line is the linear regression function using only the values at burst (circle symbols). The grey dashed line shows the linear regression using all shown symbols and the grey solid line shows the 2. order polynomial fitting all shown symbols.

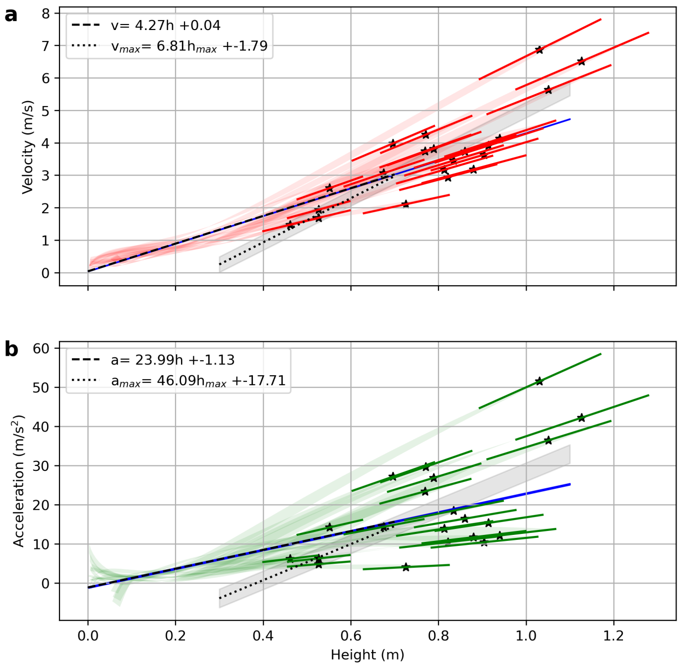

Figure S12 Comparison between bulge height and velocity (a) and acceleration (b) during the bulge growth. The color shows the timing relative to the burst time. The black stars show the final data point of each event. The dashed line is the linear regression using the entire time series of each event (all colored circles). The dotted lines show the linear regression using only the final data point of each event (black stars)

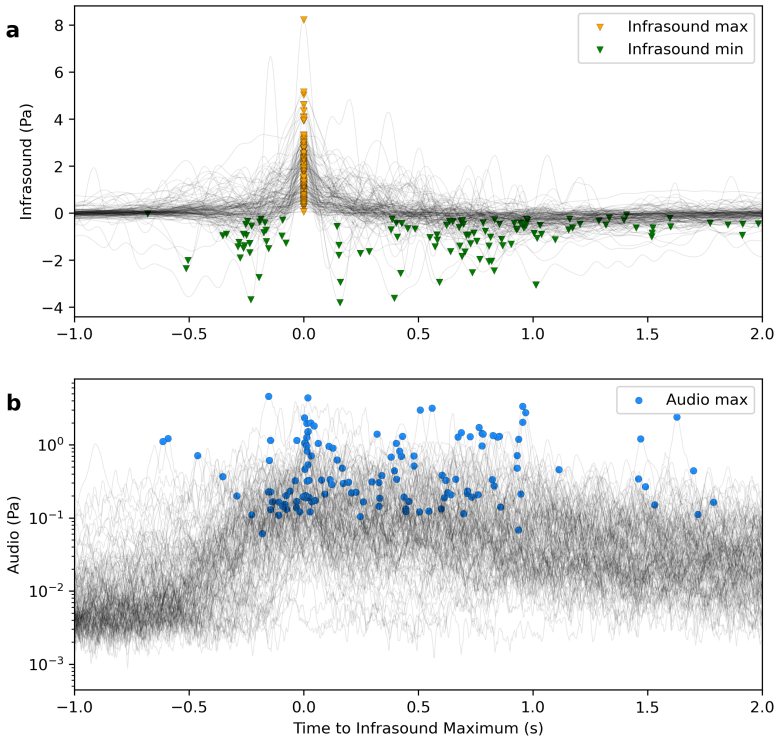

Figure S13 (a) Infrasound and (b) audio Hilbert transform envelope of events that are not showing an M-shape infrasound pattern aligned by the infrasound maximum (yellow triangles). The infrasound minimum is shown with green triangles in (a) and the audio envelope maximum is shown with blue circles in (b). There is no correlation between the parameters evident.

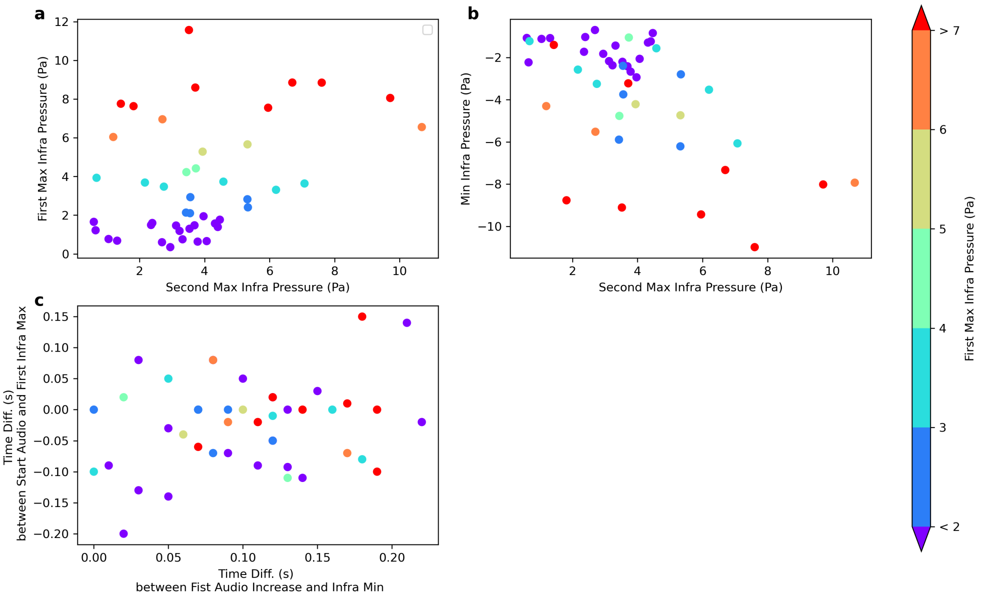

Figure S14 Mostly uncorrelated relationships between (a) the amplitude of the first and second infrasound maximum, (b) the amplitude between the second infrasound maximum and the infrasound minimum and (c) the time difference between the start of the audio and the first infrasound maximum (positive numbers mean that the audio started before the infrasound max) versus the time difference between the first significant audio increase and the infrasound minimum (positive numbers mean that the infrasound minimum happened before the audio increase). All symbols are colored by the amplitude of the first infrasound maximum.

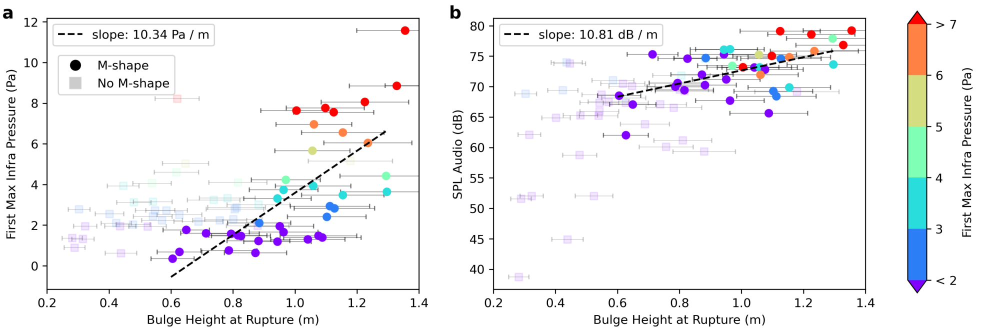

Figure S15 Scatter plots showing the relationship between maximum bulge height and the first maximum infrasound pressure (a) as well as the audio SPL over the entire time of the event (b). The transparent colored circles show events that do not exhibit the M-shape. All symbols are colored by the amplitude of the first infrasound maximum.

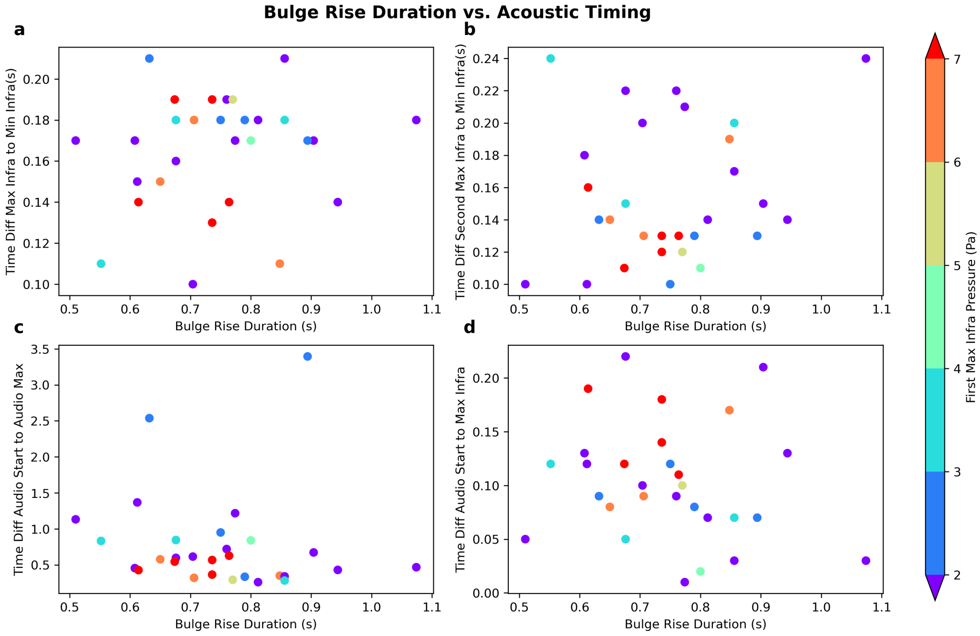

Figure S16 Relationship between the bulge rise duration and selected time differences between acoustic parameters such as the fist infrasound maximum and following minimum (a), the infrasound minimum and following second maximum (b), the start of the audio signal and its first significant audio maximum (c), and the start of the audio signal and the first infrasound maximum (d).

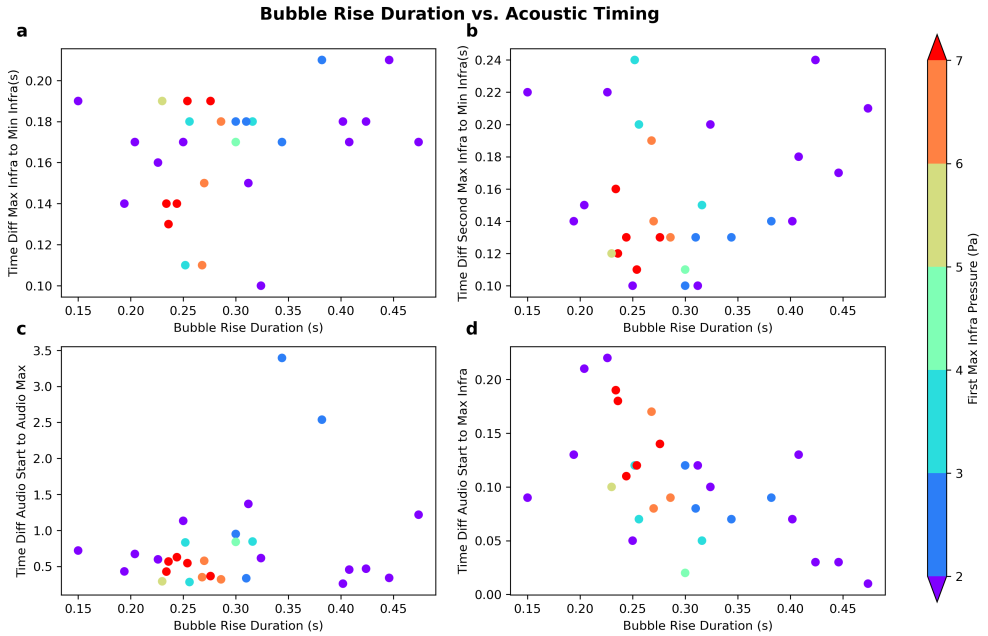

Figure S17 Relationship between the bubble rise duration and selected time differences between acoustic parameters such as the fist infrasound maximum and following minimum (a), the infrasound minimum and following second maximum (b), the start of the audio signal and its first significant audio maximum (c), and the start of the audio signal and the first infrasound maximum (d).

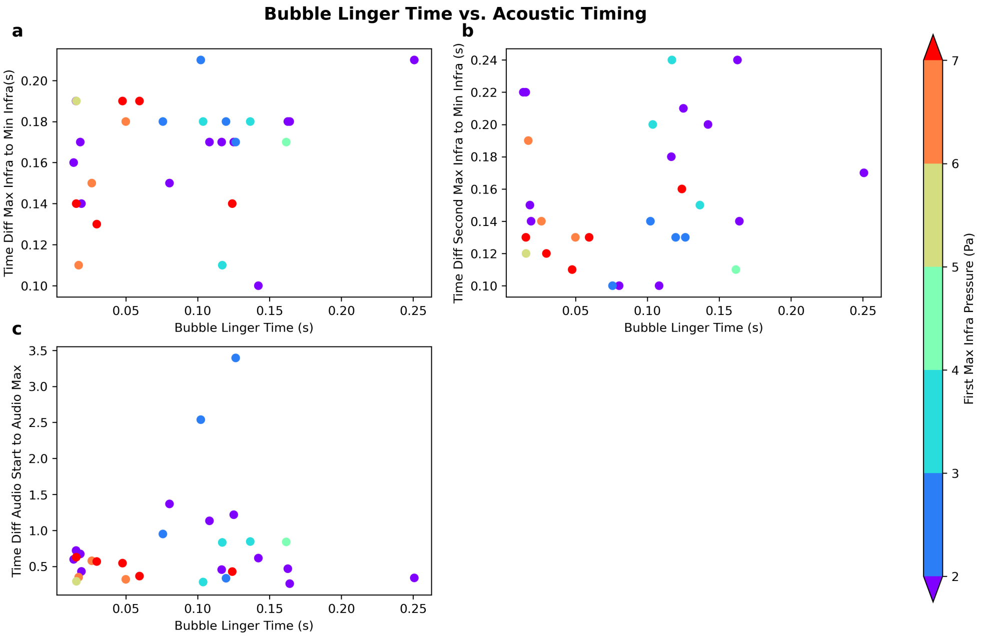

Figure S18 Relationship between the bubble linger time and selected time differences between acoustic parameters such as the fist infrasound maximum and following minimum (a), the infrasound minimum and following second maximum (b), and the start of the audio signal and its first significant audio maximum (c).

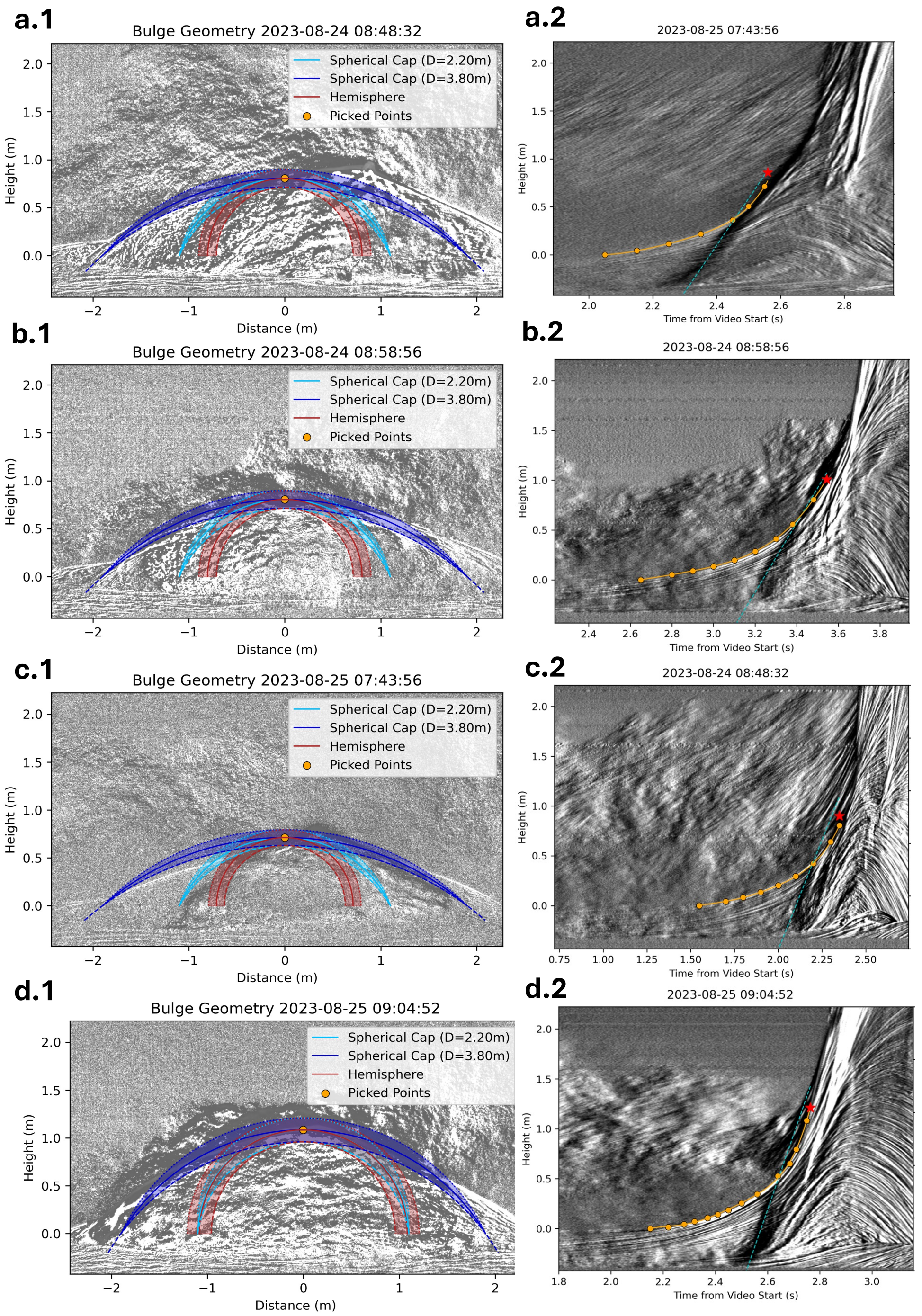

Figure S19 (a.1, b.1, c.1, d.1) Video frames of different events showing the actual bulge geometry in the background at the time of the last picked height with the different geometries of the spherical cap with the lowest (light blue) and highest (dark blue) considered diameter and the hemisphere model (red). (a.2, b.2, c.2, d.2) show kymograms of the same events. These are also the same events shown in Figure 10 in the main manuscript.

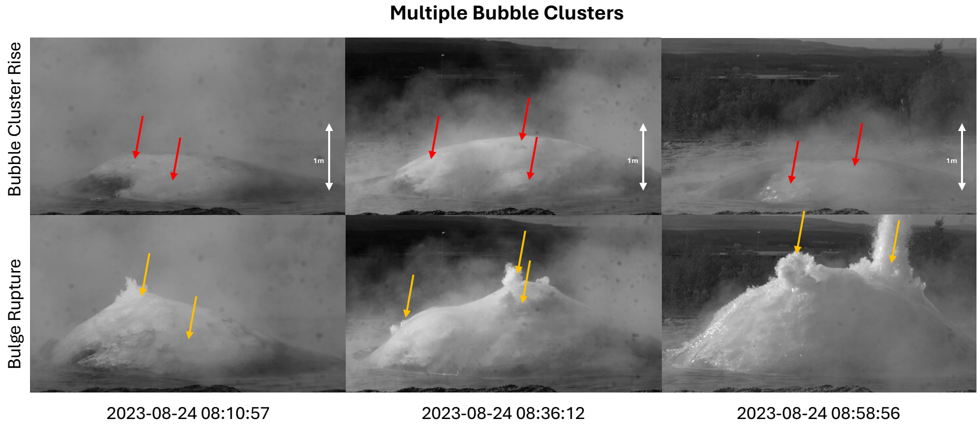

Figure S20 Three events with multiple bubble clusters and multiple rupture points. The top row is showing a frame of each event before rupture with red arrows pointing to bubble cluster. The bottom row showing a frame of each event during the beginning of the rupture with yellow arrows pointing to multiple rupture locations.

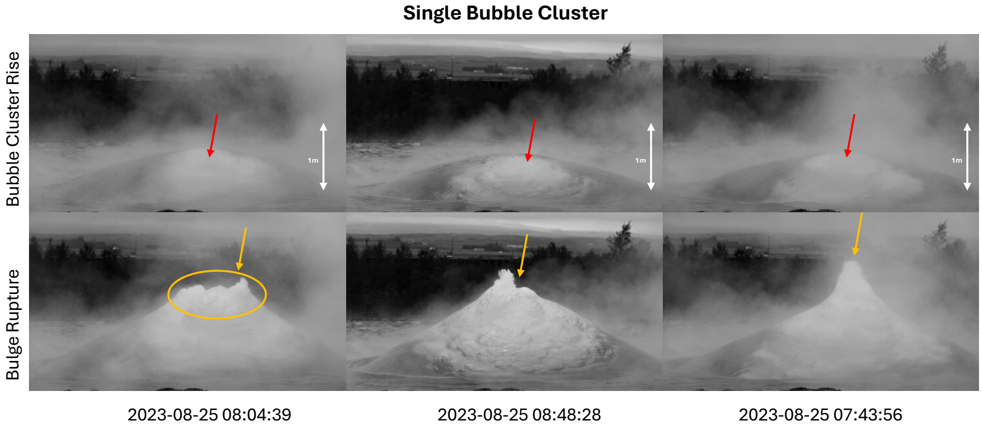

Figure S21 Three events with one main bubble cluster and one central rupture point or area. The top row is showing a frame of each event before rupture with red arrows pointing to bubble cluster. The bottom row showing a frame of each event during the beginning of the rupture with yellow arrows pointing to multiple rupture locations.
